# Consumer heterogeneity in sweet-sour preferences: Insights from sensory perception, conceptual associations, and emotional responses

**DOI:** 10.1016/j.crfs.2026.101408

**Published:** 2026-04-13

**Authors:** Jia Chen, Feifei Zhao, Fang Zhong, Juntao Kan, Huijuan Shen, Yixun Xia, Charles Spence

**Affiliations:** a Science Center for Future Foods, Jiangnan University, Wuxi, 214122, China; b School of Food Science and Technology, Jiangnan University, Wuxi, 214122, China; c Key Laboratory of Food Sensory Science and Technology, China National Light Industry, Wuxi, 214122, China; d Amway (Shanghai) Innovation and Science Co., Limited, Shanghai, 201203, China; e Crossmodal Research Laboratory, University of Oxford, Oxford, UK

**Keywords:** Consumer segmentation, Taste preference, Conceptualizations, Emotions, Binary taste mixtures

## Abstract

Characterizing the heterogeneity of consumer preferences for binary taste mixtures is important for developing targeted sweet-sour products. This study used a multi-dimensional approach to investigate the sensory, conceptual, and emotional drivers of sweet-sour preferences by examining three key questions: whether distinct preference phenotypes emerge in mixed systems, whether these differences are related to perceived intensity, and whether clusters differ in cognitive characteristics. A total of 172 females evaluated citric acid-sucrose solutions with varying intensity ratios. Hedonic responses and taste intensities were measured using a 9-point scale and the generalized Labeled Magnitude Scale (gLMS), while Check-All-That-Apply (CATA) profiling was used to capture sensory, conceptual, and emotional characteristics. Clustering based on intensity-liking correlations identified three main clusters: SWEET, SOUR, and IU (inverted U-shaped). The results showed that preference heterogeneity persists in mixed systems, although the observed patterns do not simply mirror those reported in single-taste studies. Taste intensity alone did not account for preference segmentation. Instead, the clusters differed in their conceptual and emotional profiles: the SWEET cluster favored familiar, simple, sweet-dominant experiences associated with low-arousal emotions (e.g., ‘secure’); the SOUR cluster linked intense sourness to novelty and high-arousal emotions (e.g., ‘adventurous’); the IU cluster emphasized ‘sweet-sour balance.’ The sensory-concept-emotion framework suggests that preference heterogeneity in sweet-sour mixtures is shaped not only by sensory perceptions but also by cluster-specific conceptual and emotional associations, offering useful insights for personalized flavor design and market segmentation.

## Highlights

• Three distinct preference phenotypes (SWEET, SOUR, and IU) were identified in sweet-sour taste mixtures.
• Sensory drivers of liking and optimal sweet-sour balance diverged significantly across clusters.
• Distinct ‘sensory-conceptual-emotional’ integration pathways characterized each consumer phenotype.

## Graphical abstract

**Figure:**
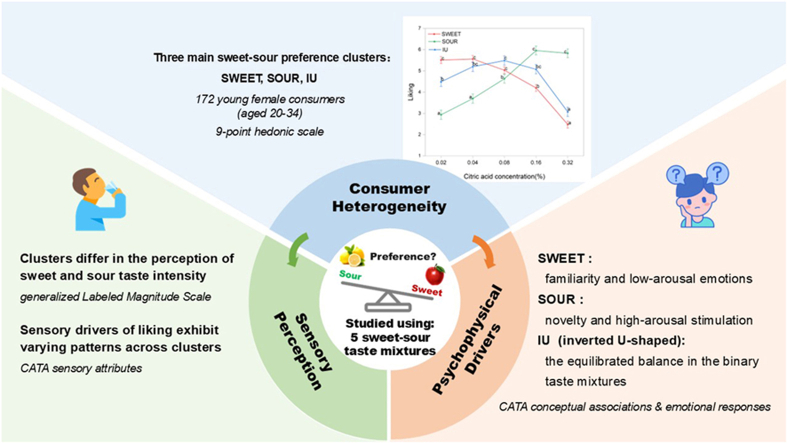

## 1 Introduction

Sweetness and sourness are two fundamental and indispensable tastes in food, widely present in fruits, flavored beverages, and culinary dishes. The perception and hedonic response to sweet and sour tastes are key factors influencing consumers’ food choice and intake (Jayasinghe et al., 2017; Liem and Russell, 2019; Sijtsema et al., 2012). Understanding hedonic responses to binary taste mixtures is of significant theoretical and practical implications for the development of novel sweet-sour flavored foods.

Heterogeneity in consumer preferences for sweetness and sourness has long been a topic of research interest. Early psychophysical studies demonstrated that the relationship between sweetness intensity and pleasantness is not simply linear. Rather, pleasantness typically peaks at intermediate concentrations (Moskowitz, 1971; Moskowitz et al., 1974). In recent years, methods for classifying sweet taste liker phenotypes have been progressively refined. Iatridi et al. (2019a) identified four classification approaches used in earlier studies: interpretation of the shape of hedonic response curves, highest preferred concentration via direct measurement, average liking ratings, and highest preference via paired comparisons. A commonly used method now defines phenotypes according to patterns of liking across stimulus intensities, which is based on intensity-liking correlations. This approach has been applied in studies of both sweet liking (Iatridi et al., 2019b; Kavaliauskaite et al., 2023; Tucker et al., 2025) and sour liking (Spinelli et al., 2024). Although different classification methods vary, the resulting hedonic patterns typically converge into discrete clusters, including increasing liking, decreasing liking, and intermediate or inverted U-shaped patterns as the intensity of sweetness or sourness increases.

However, most studies on sourness or sweetness preference phenotypes have focused on the effect of a single taste stimulus on liking, without considering interactions between the two taste qualities. Real food systems are much more complex than single-taste stimuli, and sweetness and sourness often coexist in the same product, especially in fruits, juices, and flavored beverages. Concurrently, research on sweet-sour interactions has progressed from basic qualitative descriptions of perceptual synergy and suppression (Junge et al., 2023; Keast and Breslin, 2003; Savant and McDaniel, 2004a), to quantitative studies of sweet-sour intensity relationships (Mao et al., 2019), and, more recently, to investigations of dynamic interaction patterns (Mao et al., 2025). The complexity of sweet-sour interactions suggests that the intensity-liking relationship is governed by multi-dimensional interactions, which are inherently more complex than the patterns observed in single-taste systems. Existing approaches focusing solely on single-taste intensity-liking correlations may fail to reveal the underlying preference patterns in the taste mixtures. Notably, some recent studies have begun to segment consumers in more complex food models based on the correlations between multi-taste perception and liking (Spinelli et al., 2021). Nevertheless, segmentation research focusing specifically on sweet-sour mixed systems remains limited.

Beyond interaction effects, the balance between sweetness and sourness is another important factor in understanding consumer preference. Several studies have shown that sweet-sour balance is closely related to liking, as consumers generally prefer the optimal combination of sugar and acid (Liem and de Graaf, 2004; Mao et al., 2025; McBride and Johnson, 1987). In fruit quality evaluation and production practices, the °Brix/acid ratio is often used to predict consumer acceptance of variable sweet-sour combinations (Harker et al., 2002; Jayasena and Cameron, 2008; Ross et al., 2010). However, these metrics, whether based on physical ratios or the conceptual ‘sweet-sour balance’, rely on mean hedonic ratings. Consequently, these metrics overlook the heterogeneity of taste preferences and neglect the effects of sweet-sour taste interactions, thereby limiting their ability to predict genuine consumer preferences.

With the development of sensory methods and consumer research, it has become widely recognized that liking alone cannot fully capture consumer product experience. Multidimensional information beyond liking is also needed, including consumption context, conceptual associations, and product-evoked emotions. Early food-focused studies developed systematic tools to measure food-evoked emotions, notably the EsSense Profile (King and Meiselman, 2010), and subsequent work refined these methods through improved implementation, shorter lexicons, and product-specific approaches (King et al., 2013; Nestrud et al., 2016; Richins, 1997). These “beyond liking” measurement tools have since been widely applied across diverse food systems. Research has shown that sensory cues not only shape liking but also influence emotional responses, conceptual associations, and functional conceptualizations (Gutjar et al., 2015; Ng et al., 2013; Spinelli et al., 2019a; Stolzenbach et al., 2016). Of particular relevance, emotion-based segmentation studies have revealed that different preference clusters may describe the sensory properties of products in similar terms yet exhibit distinct emotional profiles (Cardello et al., 2022; Spinelli et al., 2019b). Therefore, in sweet-sour mixed systems, examining taste intensity and liking alone may be insufficient to provide a comprehensive understanding of preference heterogeneity. “Beyond-liking” measures are also needed.

Against this backdrop, the present study aims to address three core questions. First, it investigates whether consumers exhibit preference clusters in sweet-sour mixture systems consistent with those observed in univariate systems, or if new preference phenotypes emerge. Second, the study explores whether consumers generally prefer samples characterized by balanced perceived intensities, and to what extent between-cluster differences stem from variations in perceived intensity. Third, beyond objective perceptual differences, the study examines whether preference clusters diverge at the cognitive-emotional level—specifically, whether they exhibit distinguishable emotional responses and conceptual associations.

To address these questions, a multidimensional consumer research approach was used. A total of 172 female participants evaluated five citric acid-sucrose samples with varying concentrations. Participants were classified into clusters with heterogeneous sweet-sour preference on the basis of correlations between liking and three variables (sourness intensity, sweetness intensity, and the sweet-to-sour intensity ratio). Between-cluster differences were then compared across sensory perception, emotional response, and conceptual association. Given that biological sex significantly influences taste perception (Gudziol and Hummel, 2007; Jiang and Chiang, 2023; Spence, 2019) and young adults aged 20-39 years represent a primary consumer cluster for sweet-sour products (Emanuel et al., 2012; Singh et al., 2015), the study population was limited to young females to ensure a targeted and homogeneous study population. This study is expected to complement previous work that examined sourness and sweetness separately, deepen our understanding of the preference heterogeneity in complex taste systems, and provide a scientific basis for the development of sweet-sour products targeted to specific consumer clusters.

## 2 Methods and materials

### 2.1 Participants

Participants were recruited from Jiangnan University (Wuxi, China) via a local social networking platform (Tencent QQ, Shenzhen, China). A total of 455 individuals completed an initial online screening questionnaire. The following inclusion criteria were applied to ensure a targeted and homogeneous study population: (i) female sex (self-reported); (ii) regular consumption of sweet-sour beverages (e.g., yogurt drinks or fruit juices) at least once per week; (iii) no known food allergies or taste or smell disorders; and (iv) non-smoking status. Although age was a primary consideration, no formal age-based exclusion was applied since the university-based recruitment naturally yielded a participant pool aged 20-39 years representing the primary consumer cluster for sweet-sour products. Based on these criteria, 172 female participants aged 20-34 years (mean age: 24 years) were enrolled. Detailed demographic characteristics and beverage consumption patterns are provided in Supplementary Table S1.

### 2.2 Stimuli

In real food systems, the perception of sourness and sweetness is likely to be influenced by the presence of both volatile aromas and carbonation (Stevenson et al., 1999; Yau and McDaniel, 1992). To control experimental variables and minimize the confounding effects of these additional factors, uncarbonated aqueous solutions without added volatile aromatic compounds were intentionally used in this study. Although the oral processing of nonvolatile tastants might elicit negligible olfactory responses (He et al., 2023), any such retronasal effects would be systemic across all samples and negligible relative to the primary gustatory sensations under investigation.

The overall experiment included two groups of samples. The first group consisted of five citric acid-sucrose solutions with constant sucrose (Guangzhou Fuzheng Donghai Food Co., Ltd) content (10% w/v) but differing citric acid (Weifang Ensign Industry Co., Ltd.) concentrations. To facilitate clear identification throughout the study, the five samples were labeled based on their citric acid concentrations as S0.02%, S0.04%, S0.08%, S0.16%, and S0.32%, where ‘S' denotes ‘Sample’, and the numerical value represents the citric acid concentration (w/v). Sweet-sour flavored beverages typically exhibit high levels of sweetness intensity (9-13% w/v sucrose) (Almutairi et al., 2024; Silveira Jr. et al., 2009; Ventura et al., 2011), though their sourness profiles vary considerably. The variation results in distinct sweet-to-sour intensity ratios across different products, yielding diverse product characteristics. Therefore, we fixed the sucrose concentration at 10% and altered the citric acid proportions to obtain samples with varying sensory characteristics.

Sour tastant concentrations were determined based on previous studies using sweet-sour taste mixtures (Liem and de Graaf, 2004; Savant and McDaniel, 2004b; Spinelli et al., 2024). A trained sensory panel evaluated the varying citric acid-sucrose formulations through Quantitative Descriptive Analysis. The panel consisted of 10 females (mean age: 24 years) who had extensive experience (>1.5 years) in the descriptive analysis of sweet food matrices, such as yogurt and ice cream. Prior to the formal evaluation, these panelists participated in three additional 2-h training sessions, during which they were specifically calibrated to evaluate sweet and sour intensity using aqueous reference standards. Based on the evaluation results, the five samples exhibiting significant differences in perceived sweet and sour intensities were ultimately selected.

The second group of samples comprised five concentrations of aqueous sucrose solutions (1%, 3%, 6%, 9%, and 12% w/v) and five concentrations of aqueous anhydrous citric acid solutions (0.02%, 0.04%, 0.08%, 0.16%, and 0.32% w/v). The selected concentrations spanned a broad range of taste intensities, from weak to strong. Considering the interaction effects between sour and sweet tastes in taste blending, these solutions were used to collect the participants' single-taste preferences and taste intensity perceptions.

All samples were dissolved in commercial drinking water (Huazhiwei, Jiangnan University Education and Culture Service Ltd., Wuxi, China) and stirred at room temperature (22 ± 2 °C) to ensure complete dissolution. Each sample was portioned into 15 mL aliquots in a small transparent plastic tasting cup (Jiuyuan Packaging Products Ltd., Fujian, China), labeled with three-digit randomized codes. Each participant was provided with low-sodium crackers (Aji, Malaysia) and 200 mL of drinking water as a palate cleanser.

### 2.3 Procedure

The experiment was conducted over two consecutive days. On Day 1, participants evaluated five sweet-sour mixed samples, assessing their liking, perceived taste intensity, and subjective responses to the samples. On Day 2, participants tasted a set of pure sucrose samples followed by a set of pure citric acid samples, each comprising five different concentration levels. Although the information collected in the two studies differed, the procedures for sample presentation and tasting remained consistent.

On Day 1, participants performed the evaluation of citric acid-sucrose solutions, which included five questions: a) liking ratings, b) the intensity of sweetness and sourness, c) sensory attribute perception, d) conceptual associations, and e) emotional responses.

Specifically, the 9-point hedonic scale (1 = “extremely dislike”, 9 = “extremely like”) was first used to collect participants' hedonic responses (Lim, 2011; Peryam and Pilgrim, 1957). Following the hedonic evaluation, the general Labeled Magnitude Scale (gLMS) was used to collect participants’ perceptions of the sweetness and sourness intensity of the various solutions presented. The gLMS ranged from 0 (no sensation) to 100 (strongest imaginable sensation of any kind), with intermediate anchors at 1.4 (barely detectable), 6 (weak), 17 (moderate), 35 (strong) and 51 (very strong) (Bartoshuk et al., 2003; Green et al., 1993). Prior to the test, the participants were instructed on the use of the gLMS and the 9-point hedonic scale, and then practiced rating sourness and sweetness using these scales.

Next, the sensory profile of the samples was characterized using a Check-All-That-Apply (CATA) approach. The participants were instructed to select as many attributes as they perceived in the sample from the list of sensory terms. These terms were derived from previous literature related to beverages with sour and sweet tastes, following discussion and selection by our sensory panel (An and Lee, 2024; Carbonell et al., 2007). Based on existing term selection frameworks, overly technical terms were filtered out and those strongly associated with sweet-sour taste perception were prioritized (Asih et al., 2021). The final version of the sensory lexicon comprised 15 descriptors. All sensory terms were presented to participants in simplified Chinese during testing, and Table 1 provides the original Chinese terms together with their English translations.

**Table 1:** List of sensory, conceptual, and emotional terms (in English and Chinese) used in the Check-All-That-Apply (CATA) task.

Factor	Terms
Sensory	Sweet, Sour, Astringent, Bitter, Sour-then-sweet (先酸后甜的), Sweet-then-sour (先甜后酸的), Short-lived sweet (甜味消失快的), Long-lasting sweet (甜味持久的), Short-lived sour (酸味消失快的), Long-lasting sour (酸味持久的), Tongue numbing (舌头麻木的), ‘Sets-teeth-on-edge’ (牙齿酸软的), Sharp (口感尖锐的), Smooth (口感柔和的), Bland (寡淡的)
Conceptual	Novel (新奇的), Ordinary (普通的), Surprising (令人惊讶的), Boring (乏味的), Fantastic (奇妙的), Simple (简单的), Complex (复杂的), Direct (直接的), Obscure (难以描述的), Balanced (平衡的), Shattered (分离的), Short-lived (短暂的), Elaborated (丰富的), Unique (独特的), Familiar (熟悉的)
Emotional	Warm (温暖的), Bored (无聊的), Disgusted (令人恶心的), Nostalgic (怀旧的), Understanding (能理解的), Wild (野蛮的), Good Natured (性情温和的), Joyful (心情愉快的), Interested (感兴趣的), Happy (幸福的), Free (自由的), Guilty (愧疚的), Mild (温和的), Satisfied (满意的), Pleasant (符合心意的), Tame (温顺的), Loving (钟爱的), Secure (安心的), Active (活跃的), Enthusiastic (充满热情的), Worried (焦虑的), Aggressive (好争斗的), Calm (平静的), Adventurous (有冒险精神的), Good (高兴的)

The fourth and fifth questions similarly used the CATA approach to capture participants’ conceptual associations and emotional responses after consuming the sample. Based on the existing literature concerning conceptual analysis and sweet-sour flavored beverage consumption, 15 conceptual terms were discussed and selected (An and Lee, 2024; Lévy et al., 2006; Stolzenbach et al., 2016). The emotion lexicon was directly sourced from EsSense25 (Nestrud et al., 2016), but all terms were presented in simplified Chinese during testing. The Chinese terms used in the test and their English equivalents are listed in Table 1.

On Day 2, participants evaluated two sets of single taste solutions, each containing five concentrations. Participants were asked to rate their liking for these samples using the 9-point hedonic scale and evaluate the sweetness or sourness intensity of these samples using the gLMS. To minimize sensory fatigue and carry-over effects of the sour samples, all participants evaluated the sucrose samples first, followed by a 5-min break before evaluating the citric acid samples.

During the test, the participants were instructed to rinse with the entire 15-mL aliquot for 5 s before swallowing. Subsequently, they assessed their preference for this sample, followed by evaluating the perceived intensity of sour or sweet taste. To ensure full gustatory recovery and alleviate cognitive monotony, each participant was provided with low-sodium crackers (Aji, Malaysia) and 200 mL of water as a palate cleanser, and was required to rest for exactly 2 min before proceeding to the next sample.

All of the samples were sequentially presented to participants. The sample presentation order was balanced across participants using a Williams Latin Square design. The presentation order of the CATA terms was dynamically executed using the built-in randomization algorithm of the Tencent Wenjuan survey platform. Data were collected through this platform. Evaluations were conducted in a standardized sensory laboratory (Jiangnan University, Wuxi, China) designed in accordance with ISO 8589:2007. All tests were performed in individual booths under controlled white light to ensure independent judgment and visual consistency.

Ethical approval for involving human participants in this study was granted by the Jiangnan University Medical Ethics Committee (Reference Number: JNU20221201IRB03). Participants in the experiment received gifts as compensation.

### 2.4 Statistical analysis

Adapted from the approach of Green et al. (2010), who compared gLMS-based taste intensity ratings across taste qualities to characterize taste dominance in mixtures, we used the sweet-to-sour intensity ratio (sweet/sour ratio) as a descriptive index of the relative dominance of sweetness and sourness in each sample. We defined the classification thresholds based on the perceptual patterns observed in the current stimulus set. Samples with a ratio below 1.0 were classified as sour-dominant; samples with a ratio between 1.0 and 2.0 were classified as having approximately balanced sweet and sour intensities; and samples with a ratio above 2.0 were classified as sweet-dominant. Notably, these thresholds are based on our empirical data and provide a preliminary framework for characterizing taste dominance in mixed systems. However, more universal threshold standards remain to be further refined by future research.

Pearson correlation coefficients were calculated for each participant between liking ratings and sweetness intensity, sourness intensity, and sweet/sour ratios, respectively. K-means clustering analysis was performed on the individual correlation coefficients (r-values) to identify four consumer clusters, each characterized by a specific sensory-liking pattern (Iatridi et al., 2019b; Spinelli et al., 2021). To ensure the stability and optimal convergence of the clustering solution, the K-means algorithm was initialized with multiple random starting points and configured for up to 500 iterations (Wajrock et al., 2008). Four participants were excluded from the cluster analysis because they showed zero variance in the evaluation of at least one sensory attribute (i.e., they rated all samples with the same score), making it impossible to calculate their correlation coefficients.

A mixed-model analysis of variance (ANOVA) was applied to evaluate the effects of consumer cluster, sample, and their interaction (Cluster × Sample) on liking patterns and sensory intensity ratings (sweetness and sourness). In these models, liking scores were treated as the dependent variables; cluster, sample, and their interaction were defined as fixed effects, while participants (nested within each cluster) were considered random effects. The significance of fixed effects was assessed using Type III F-tests. Satterthwaite's approximation method was used to generate the statistical tests. When a significant Cluster × Sample interaction was detected, simple effects of sample within each cluster were further examined within the same mixed-model framework. Pairwise comparisons among samples within each cluster were then conducted based on least-squares (LS) means, with Tukey's post hoc test for multiple testing. A significance level of α = 0.05 was used. The same approach was applied to analyze the intensity evaluations of citric acid solutions, sucrose solutions, and their mixtures across different consumer clusters.

CATA binary data from the sensory, conceptual, and emotional blocks were analyzed for the total participant sample and separately for each consumer cluster. Cochran's Q test was performed for each term to evaluate significant differences in citation frequencies among samples (α = 0.05). When significant differences were identified, McNemar's test was applied to conduct the pairwise comparisons between samples specifically for that significant term (Ares and Jaeger, 2015; Meyners et al., 2013). Correspondence Analysis (CA) was conducted on the sample × term contingency tables using chi-square distance. Terms with an overall citation frequency <10% or those lacking significant discriminating power (p > 0.05) were excluded from the Correspondence Analysis (Meyners et al., 2013; Vidal et al., 2015). The results were visualized using asymmetric row-principal biplots, where samples were plotted in principal coordinates and terms in standard coordinates.

To determine the impact of sensory attributes and subjective responses (conceptual and emotional) on the liking ratings, penalty/lift analysis was used by calculating the mean impact of each CATA term, with a threshold value of 10% citation frequency, on overall liking (Cardello et al., 2022; Jaeger et al., 2024; Meyners et al., 2013). By interpreting the positive and negative effects of CATA term selection on liking ratings, this analysis elucidated the objective and subjective drivers of liking across different consumer clusters.

To integrate the sensory, conceptual, and emotional characteristics of the samples, multiple factor analysis (MFA) was performed (Escofier and Pagès, 1994; Spinelli et al., 2019a). For each consumer cluster, terms that significantly discriminated among samples were selected. Citation frequencies of sensory, conceptual, and emotional terms were used as active variables, while liking was included as a supplementary variable (Giacalone and Jaeger, 2021). RV coefficients were calculated across all blocks to assess the correlations between different aspects (Martins et al., 2021; Robert and Escoufier, 1976).

All analyses were performed using XLSTAT (XLSTAT 2019.2.2, Addinsoft, USA).

## 3 Results

### 3.1 Sensory characterization and intensity profiles of sweet-sour mixtures

**Fig. 1:**
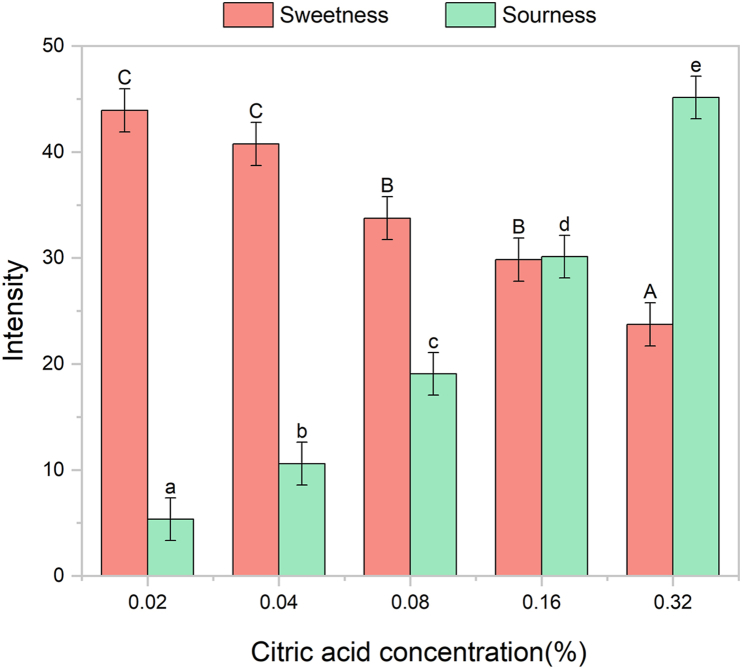
Mean intensity ratings (±95% Confidence Intervals) for sweetness (red) and sourness (green) across five sweet-sour mixtures with varying citric acid concentrations (w/v). Sucrose concentration was constant at 10% (w/v) for all samples. Different letters indicate significant differences among samples (p < 0.05) based on two-way mixed-model ANOVA followed by Tukey's post hoc test.

A total of five samples with distinct sensory characteristics were evaluated in the study. As intended, while the sucrose concentration was maintained at 10% across all samples, the citric acid concentration was incrementally increased from 0.02% to 0.32%. As illustrated in Fig. 1, sourness intensity exhibited a significant increase with citric acid concentration, with LS means rising from 5.36 (S0.02%) to 45.14 (S0.32%). Despite the constant sucrose content, sweetness intensity decreased significantly from 43.94 (S0.02%) to 23.74 (S0.32%), possibly due to the mixture suppression of sweetness by sourness (Green et al., 2010; Junge et al., 2020; Savant and McDaniel, 2004a, Savant and McDaniel, 2004b).

Sweetness and sourness intensity ratings further allowed the relative dominance of the two taste qualities to be summarized using the sweet/sour ratio defined in the Methods. Based on this ratio, S0.32% was classified as sour-dominant (sweet/sour <1), S0.08% and S0.16% were characterized by approximately comparable sweet and sour intensities (1 < sweet/sour <2), and S0.02% and S0.04% were categorized as sweet-dominant (sweet/sour >2).

**Fig. 2:**
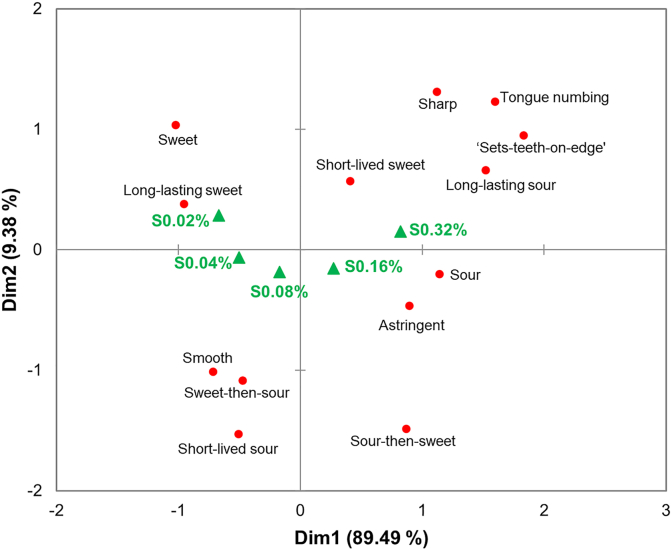
The biplot from Correspondence Analysis based on the first two dimensions, showing the distribution of sensory attributes across five sweet-sour mixtures with varying citric acid concentrations (S0.02%, S0.04%, S0.08%, S0.16%, S0.32%, w/v). Green triangles represent samples, and red circles represent sensory attributes.

In addition to variations in sweet and sour intensity, the Correspondence Analysis plots indicated that the five samples were spatially differentiated by their dynamic sweet-sour characteristics and mouthfeel attributes. As visualized in Fig. 2, the sweet-dominant sample S0.02% was strongly associated with a ‘smooth’ mouthfeel and ‘long-lasting sweetness.’ Compared to S0.02%, S0.04% displayed a marginal increase in sourness intensity; it maintained its association with sweet dominance but shifted closer to the ‘sweet-then-sour’ dynamic transition. Although samples S0.08% and S0.16% reached comparable sweet and sour intensity levels, they occupied distinct regions in the biplot primarily due to their temporal profiles. S0.08% was tightly linked to the ‘sweet-then-sour’ pattern, whereas S0.16% was positioned near the ‘sour-then-sweet’ sequence. Finally, the sour-dominant S0.32% was predominantly associated with ‘sour’ or ‘long-lasting sourness,’ and co-located with a ‘sharp’ mouthfeel and specific somatosensory responses, such as ‘sets-teeth-on-edge’ and ‘tongue numbing.’

### 3.2 Identification and segmentation of sweet-sour preference phenotypes

**Fig. 3:**
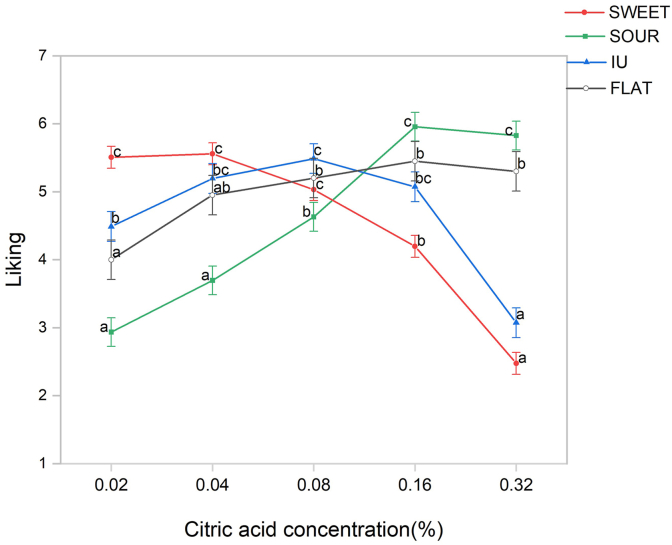
Mean liking scores (±) SE of four consumer clusters for five sweet-sour mixtures. Different letters indicate significant differences among samples within each cluster (p < 0.05) based on two-way mixed-model ANOVA followed by Tukey's post hoc test.

Cluster analysis was conducted using individual correlation coefficients. These coefficients represented the relationship between liking and the sweet/sour ratio, sourness intensity, and sweetness intensity. Four consumer clusters with distinct sensory-liking patterns were identified and designated as ‘SWEET,’ ‘SOUR,’ ‘IU’ (Inverted U-shape), and ‘FLAT’ based on their specific response profiles (Table 2 and Fig. 3). The mixed-model ANOVA for liking revealed a significant sample × cluster interaction effect (F = 33.15, p < 0.001), statistically validating these divergent sensory-liking patterns among the four clusters.

**Table 2:** Correlation coefficients between mean liking scores and the sweet/sour ratio, sweetness intensity, and sourness intensity for the four consumer clusters.

Cluster	Sweetness/Sourness	Sweetness	Sourness	Cluster size (n)
SWEET	0.524	0.486	−0.748	61
SOUR	−0.781	−0.779	0.675	46
IU	−0.087	−0.351	−0.386	41
FLAT	−0.416	0.095	0.280	20

The SWEET cluster (n = 61, 36.3%) exhibited a strong positive correlation between liking and the sweet/sour ratio, showing a preference for sweet-dominant samples. In this cluster, liking ratings declined significantly (from 5.56 to 2.48) as the sweet/sour ratio decreased. Conversely, the SOUR cluster (n = 46, 27.4%) demonstrated a strong negative correlation with the sweet/sour ratio (i.e., a positive correlation with sourness intensity). Their liking scores increased from 2.94 to 5.96 as the samples became more sour-dominant (meaning the sweet/sour ratio decreased). In the IU cluster (n = 41, 24.4%), the correlation between liking and the sweet/sour ratio was weak (−0.087). However, it exhibited negative correlations with both sourness and sweetness intensities, with mean coefficients approximating −0.5. Liking scores for the IU cluster followed a monotonic increase, peaking at a citric acid concentration of 0.08% before declining, forming an inverted U-shaped preference pattern. Finally, the FLAT cluster (n = 20, 11.9%) displayed weak correlations between liking and taste intensities, with liking scores remaining relatively stable across all samples (ranging from 4.00 to 5.45). Subsequent analyses focused on the three clusters with distinct, directional preferences (SWEET, SOUR, and IU).

### 3.3 Variations in basic taste perception and mixture suppression across clusters

**Fig. 4:**
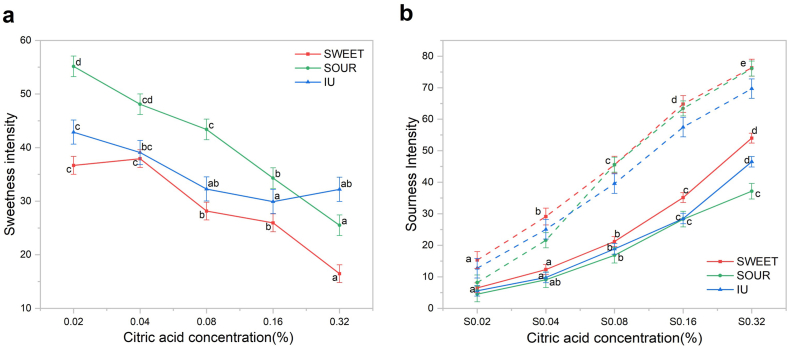
Mean intensity ratings (±SE) for sweetness (a) and sourness (b) perceived by the SWEET, SOUR, and IU clusters. For sourness (b), solid lines represent perceived intensity in sweet-sour mixtures, while dashed lines represent pure citric acid solutions. Different letters indicate significant differences among samples within each cluster (p < 0.05) based on two-way mixed-model ANOVA followed by Tukey's post hoc test.

The mixed-model ANOVA revealed significant differences in taste intensity ratings (Fig. 4a and b). A main effect of concentration, cluster, and their interaction (all p < 0.001, Table 3) were all evident for sweetness and sourness intensities. This suggests that different consumer clusters exhibited distinct intensity responses as the sample's sweet/sour ratio varied.

**Table 3:** Analysis of variance (Type III Tests) for the effects of citric acid concentration, consumer cluster, and their interaction on sweetness and sourness intensity ratings in sweet-sour mixtures.

Main effect	Num DF	Den DF	F	Pr > F
Sweetness intensity				
Concentration	4	690.9	50.097	<0.001
Cluster	3	170.6	6.252	<0.001
Concentration∗Cluster	12	690.9	3.492	<0.001
Sourness Intensity				
Concentration	4	666.2	189.762	<0.001
Cluster	3	166.8	4.414	0.005
Concentration∗Cluster	12	666.2	3.052	<0.001

Regarding sweetness intensity, the SOUR cluster reported higher mean intensity compared to the SWEET and IU clusters. At the lowest concentration of 0.02%, the SOUR cluster rated sweetness intensity at 55.2 (‘very strong’), significantly higher than the mean score reported by the SWEET cluster (36.7). For sourness, average ratings across clusters did not differ at the concentration under 0.08%. Sourness intensity was weak at 0.02% (3.3-6.5), moderate-to-weak at 0.04% (9.0-12.3), and moderate at 0.08% (16.8-21.2). However, at high concentrations (0.16%, 0.32%), the SWEET cluster reported significantly higher sourness intensity with the average rating for S0.32% exceeded 51 (‘very strong’). In contrast, the SOUR cluster perceived the mean sourness intensity of S0.32% as only 37.2.

The perceptual differences in the sweet-sour mixtures may relate to individual variations in basic taste perception and preference. Consequently, basic taste phenotypes were examined in pure taste samples across sucrose and citric acid gradients. Table 4 shows significant sample × cluster interaction for sweetness liking (p < 0.001) and sweetness intensity (p = 0.005) perceived in sucrose solutions. Conversely, no effect of interaction was found for sourness liking (p = 0.360) and sourness intensity (p = 0.110) in citric acid solutions, indicating that sourness perception patterns ware similar across clusters. Most participants exhibited negative hedonic responses to samples containing only citric acid.

**Table 4:** Analysis of variance (Type III Tests) for the effects of concentration, consumer cluster, and their interaction on intensity and liking ratings in pure sucrose and citric acid solutions.

Main effect	Num DF	Den DF	F	Pr > F
Sweetness Liking				
Concentration	4	666.2	189.762	<0.001
Cluster	3	166.8	4.414	0.005
Concentration∗Cluster	12	666.2	3.052	<0.001
Sweetness Intensity				
Concentration	4	680.1	569.687	<0.001
Cluster	3	168.9	2.496	0.062
Concentration∗Cluster	12	680.1	2.370	0.005
Sourness Liking				
Concentration	4	656.1	147.158	<0.001
Cluster	3	164.0	10.551	<0.001
Concentration∗Cluster	12	656.1	1.097	0.360
Sourness Intensity				
Concentration	4	696.2	392.918	<0.001
Cluster	3	171.7	7.140	<0.001
Concentration∗Cluster	12	696.2	1.525	0.110

Comparisons with the pure citric acid samples further indicate that sweetness suppressed sourness perception across all the clusters, although the extent of this suppression varied depending on the cluster. At a citric acid concentration of 0.32%, the perceived sourness intensity of the citric acid-sucrose mixtures was lower than that of the pure citric acid solutions. This reduction was greatest in the SOUR cluster, in which sourness ratings decreased by 51% (from 76.07 to 37.15), followed by the IU cluster, with a 33% reduction (from 69.68 to 46.45), and was smallest in the SWEET cluster, with a 26% reduction (from 76.33 to 56.00). Consequently, while sweetness suppressed sourness perception for all consumers, this suppressive effect was stronger in the SOUR cluster than in the SWEET cluster. Although absolute sweetness ratings differed due to variations in their pure-sucrose baselines, the relative reduction in perceived sweetness intensity within the mixtures was comparable across all groups as citric acid increased from 0.02% to 0.32%. Across this concentration gradient, both the SWEET and SOUR clusters experienced an approximately 50% reduction in perceived sweetness.

### 3.4 Sensory, conceptual, and emotional drivers of sweet-sour liking

#### 3.4.1 Sensory drivers of sweet-sour liking

**Fig. 5:**
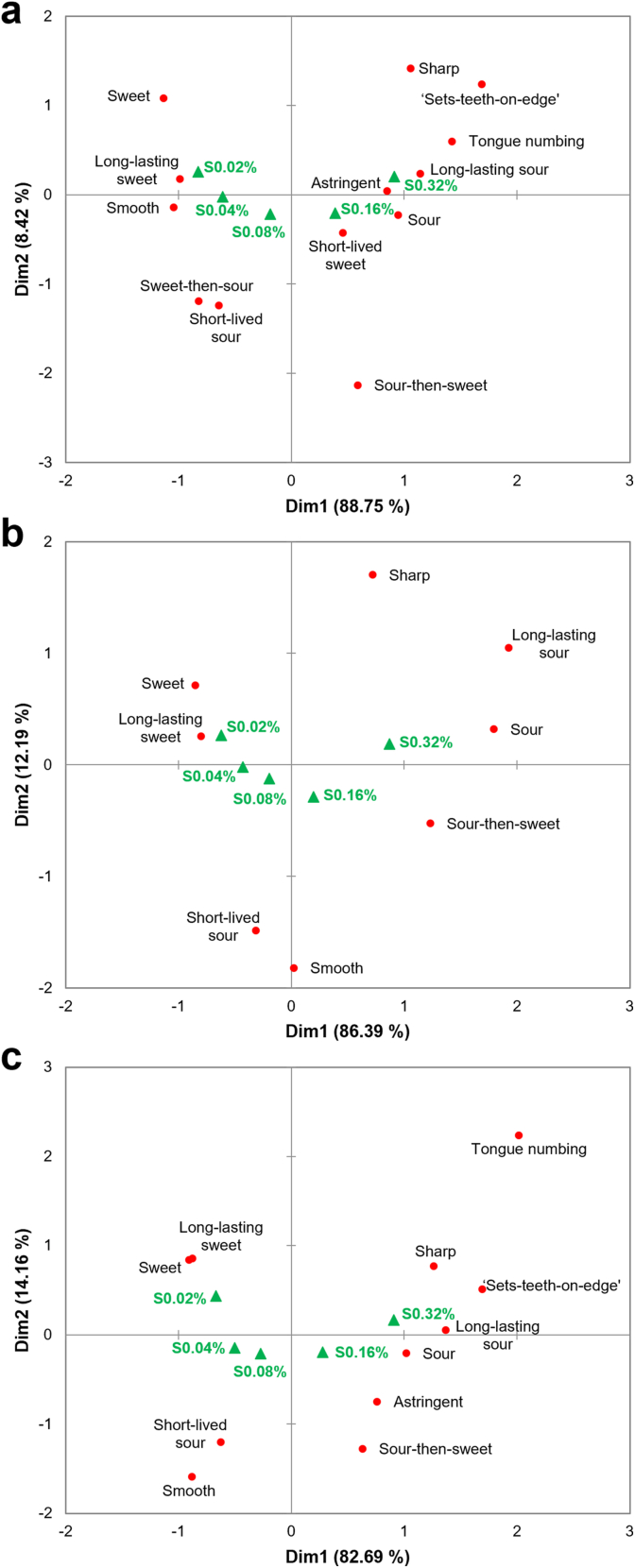
Biplots from Correspondence Analysis based on the first two dimensions, showing the distribution of samples and CATA sensory descriptors for the SWEET (a), SOUR (b), and IU (c) clusters. For each cluster, only descriptors with a citation frequency >10% and significant discrimination amongst the samples (p < 0.05) were included in the analysis.

CATA was used to evaluate dynamic taste experiences and mouthfeel attributes. Fig. 5a–c illustrated the sensory perception maps for the three consumer clusters. Biplots were constructed using the first two dimensions of Correspondence Analysis. These plots included sensory attributes that significantly discriminated among samples within each cluster (citation frequency >10%, p < 0.05).

Comparing the biplots for the three consumer clusters (Fig. 5a–c), the distribution of basic taste dominance and dynamic sensory transitions remained highly consistent with the overall cohort map (Fig. 2). However, pronounced cluster-specific variations emerged regarding the associations of mouthfeel attributes. Attributes associated with high acidity were infrequently cited by the SOUR cluster (citation frequency <10%). Consequently, they tended to associate S0.16% more closely with a ‘smooth’ mouthfeel and plotted S0.32% relatively far from extreme ‘sharpness’ or discomfort terms. In contrast, the biplots for the SWEET and IU clusters showed S0.16% and S0.32% clustering tightly with a ‘sharp’ mouthfeel and descriptors such as ‘sets-teeth-on-edge.’

Penalty/lift analysis quantified the impact of specific sensory attributes on liking (Table 5). The results confirmed fundamental differences in sensory drivers among the three clusters. Consistent with our hypothesis, sweetness-related attributes (e.g., ‘sweet’) were the primary lift factor for the SWEET cluster (+1.871). However, these attributes reduced the liking for the SOUR cluster by approximately 2.1 points. In contrast, sourness-related attributes, such as ‘sour,’ significantly increased liking for the SOUR cluster (+1.129) but decreased it for the SWEET cluster (−1.153). Additionally, ‘smooth’ was a significant lift factor for liking across all three clusters, with a mean lift exceeding 1.5 points. For the SWEET and IU clusters, the presence of ‘sets-teeth-on-edge’ (−2.399 for SWEET, −1.588 for IU) and ‘sharp’ mouthfeel (−2.034 for SWEET, −2.030 for IU) significantly reduced liking.

**Table 5:**
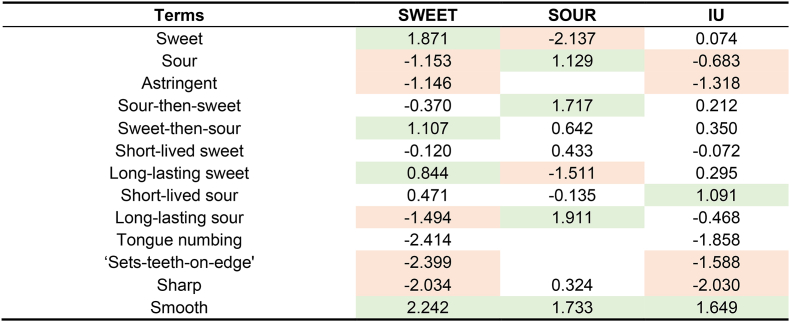
Penalty/lift analysis of the impact of sensory attributes on liking scores across consumer clusters.

Liking was measured on a 9-point hedonic scale. Values represent the mean impact on liking. Green shading indicates that the presence of the attribute significantly increased liking (p < 0.05), while red shading indicates a significant decrease (p < 0.05). Blank cells indicate descriptors with a citation frequency <10% for that cluster.

#### 3.4.2 Conceptual associations and the role of critical concepts

**Fig. 6:**
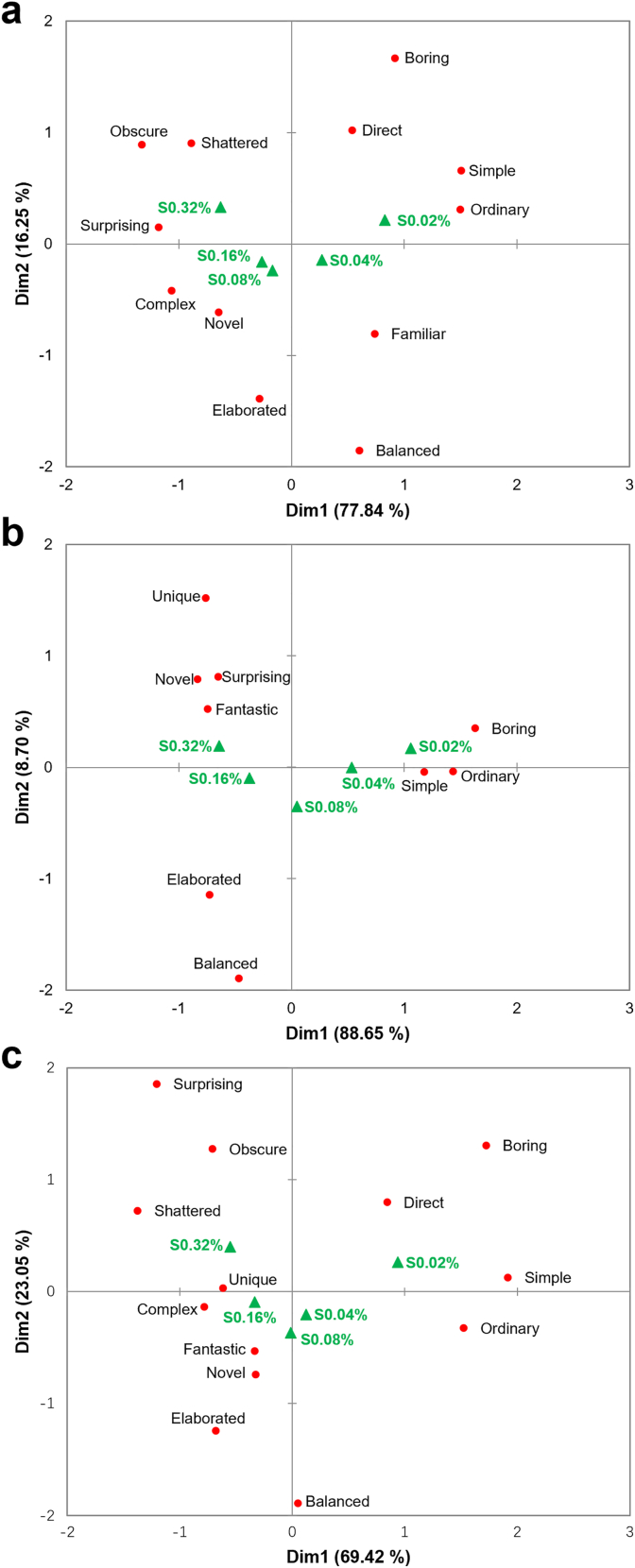
Biplots from Correspondence Analysis based on the first two dimensions, showing the distribution of samples and CATA conceptual associations for the SWEET (a), SOUR (b), and IU (c) clusters. For each cluster, only descriptors with a citation frequency >10% and significant discrimination among samples (p < 0.05) were included in the analysis.

Fig. 6 illustrates the conceptual associations identified by the consumer clusters after tasting samples with various sweet/sour ratios. Correspondence Analysis was performed on the first two dimensions based on concepts that significantly discriminated among samples within each cluster (citation frequency >10%, p < 0.05). For the SWEET, SOUR, and IU clusters, sample differentiation primarily occurred along the X-axis. The first principal component explained 77.84%, 88.65%, and 69.42% of the variance for each cluster, respectively.

Overall, consumer clusters with distinct sweet-sour liking patterns shared several common associations for the samples. For example, sweet-dominant samples (S0.02%, S0.04%) were positioned close to concepts such as ‘simple’ and ‘boring.’ The sour-dominant sample (S0.32%) was linked to terms such as ‘unique’ and ‘surprising.’ S0.08% and S0.16%, which featured dynamic sweet-sour transitions, were perceived as relatively ‘elaborated.’

Despite these similarities, cluster-specific differences emerged regarding the association of the key concept ‘balanced.’ For the SWEET cluster, ‘balanced’ was linked to S0.02% and S0.04%, and was positioned alongside terms such as ‘familiar’ and ‘simple.’ In contrast, the SOUR cluster associated ‘balanced’ with S0.32%, which was also characterized as ‘novel’ and ‘surprising.’ The IU cluster used ‘balanced’ to describe S0.08% and S0.16%. These two samples possessed sweet/sour ratios near 1 and offered a more ‘elaborated’ taste experience as compared to the samples with extreme taste dominance.

Penalty/lift analysis (Table 6) indicated that conceptual associations exerted similar driving impacts on liking across the three consumer clusters. When samples were characterized as ‘novel,’ ‘balanced,’ ‘elaborated,’ ‘unique,’ or ‘familiar,’ liking increased to varying degrees. Selection of the term ‘balanced’ was associated with a liking lift exceeding 1.5 scale points for the SOUR, SWEET, and IU clusters. Conversely, the concept ‘obscure’ significantly suppressed liking for the SWEET and IU clusters, decreasing by more than 2 scale points compared to those samples that did not leave an ‘obscure’ impression.

**Table 6:**
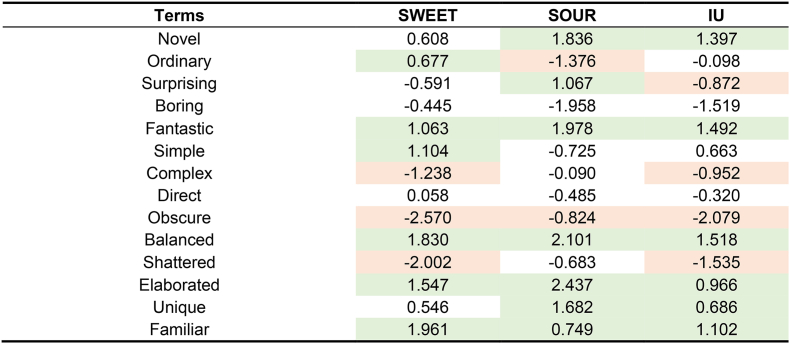
Penalty/lift analysis of the impact of conceptual associations on liking scores across consumer clusters.

Liking was measured on a 9-point hedonic scale. Values represent the mean impact on liking. Green shading indicates that the presence of the attribute significantly increased liking (p < 0.05), while red shading indicates a significant decrease (p < 0.05).

To identify cluster-specific drivers of liking, conceptual terms with a mean impact exceeding 1.5 scale points were compared (Table 6). Beyond ‘balanced’ and ‘shattered,’ the SWEET cluster was primarily influenced by sample ‘elaboration’ (1.547) and ‘familiarity’ (1.961). The SOUR cluster was more sensitive to whether samples were perceived as ‘boring’ (−1.958) or possessed ‘novelty’ (1.836). For the IU cluster, ‘balanced’ was the primary driver. The impacts of all other conceptual terms for this cluster remained below 1.5 scale points.

#### 3.4.3 Emotional responses and their association with sweet-sour liking

**Fig. 7:**
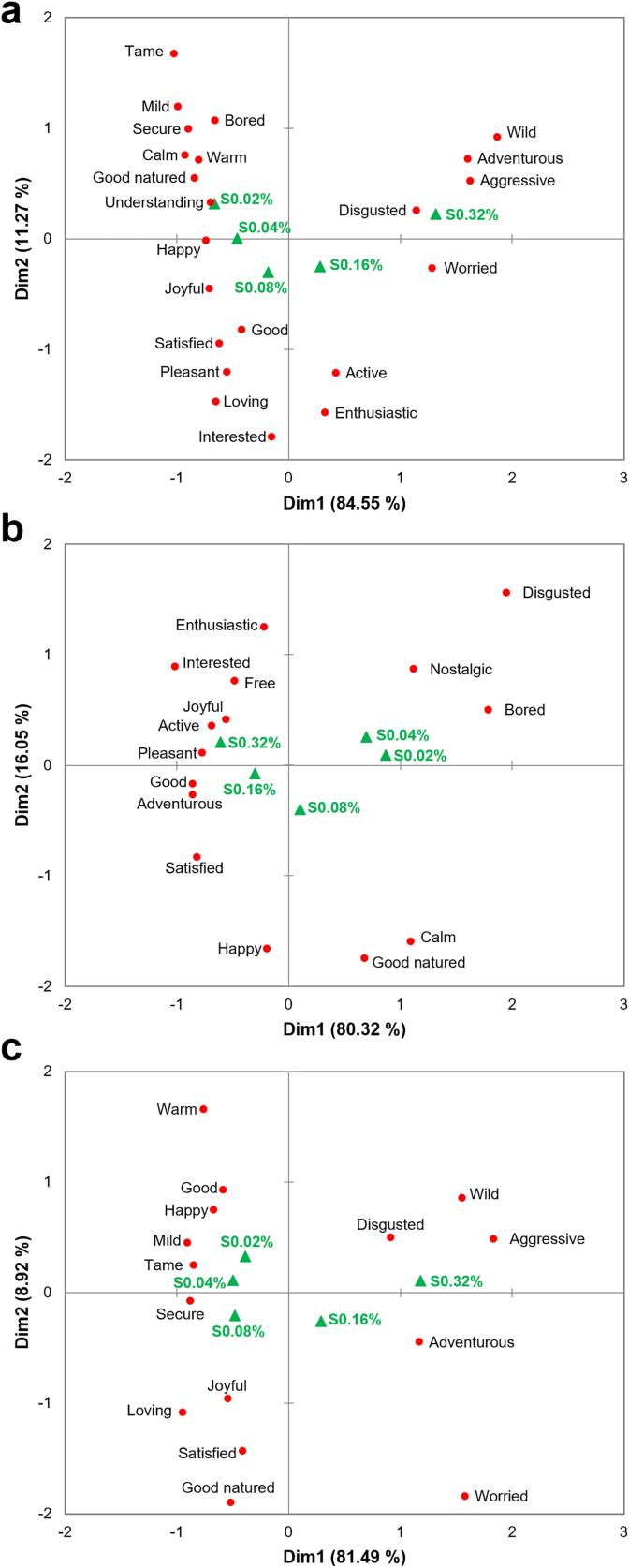
Biplots from Correspondence Analysis based on the first two dimensions, showing the distribution of samples and CATA emotional responses for the SWEET (a), SOUR (b), and IU (c) clusters. For each cluster, only descriptors with a citation frequency >10% and significant discrimination among samples (p < 0.05) were included in the analysis.

Fig. 7 presents the emotional responses of the consumer clusters after tasting, focusing on terms that discriminated significantly amongst samples. No significant cluster-specific differences were observed along the arousal dimension. All clusters reported higher frequencies of high-arousal emotions, such as ‘active’ and ‘enthusiastic’ for S0.32%. Conversely, low-arousal emotions, such as ‘warm’ and ‘mild’ were more frequently associated with S0.02% and S0.04% across all clusters.

In contrast, significant cluster-specific variations emerged along the valence dimension. The SWEET cluster associated S0.02% and S0.04% with positive emotions, including ‘happy’ and ‘joyful.’ While the SOUR cluster linked these samples to negative emotions, such as ‘disgusted’ and ‘worried.’ For S0.32%, the SOUR cluster reported positive emotions, such as ‘pleasant’ and ‘interested.’ However, the SWEET and IU clusters reported feelings of being ‘disgusted’ in response to this sample.

Penalty/lift analysis (Table 7) was conducted in order to evaluate the impact of emotional responses on liking. Among the terms with significant effects (p < 0.05), positive-valence emotions generally functioned as positive drivers of liking. When participants associated samples with terms such as ‘pleasant,’ ‘happy,’ ‘satisfied,’ or ‘joyful,’ liking scores increased by an average of more than 1.5 scale points. Conversely, selection of the negative-valence term ‘disgusted’ was associated with a decrease in liking of 2.898 points for the SOUR cluster, 2.827 points for the SWEET cluster, and 2.988 points for the IU cluster.

**Table 7:**
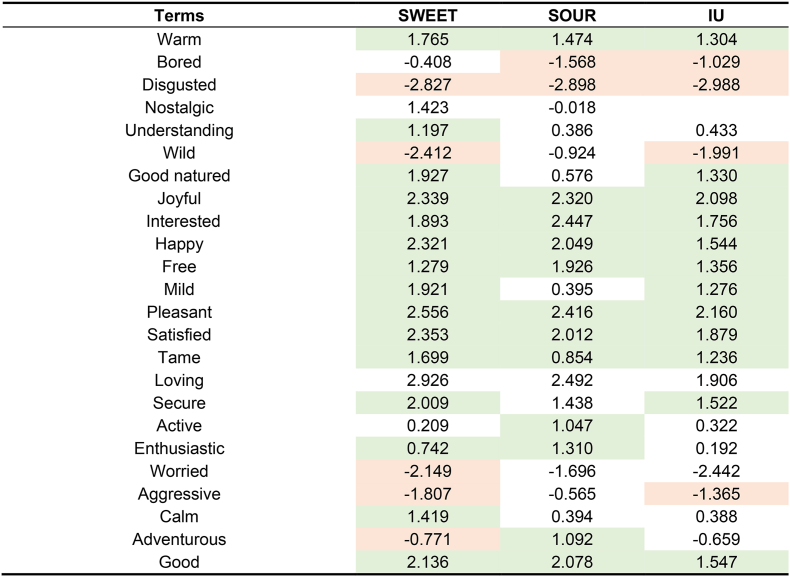
Penalty/lift analysis of the impact of emotional responses on liking scores across consumer clusters.

Liking was measured on a 9-point hedonic scale. Values represent the mean impact on liking. Green shading indicates that the presence of the attribute significantly increased liking (p < 0.05), while red shading indicates a significant decrease (p < 0.05). Blank cells indicate descriptors with a citation frequency <10% for that cluster.

Additionally, certain positive, low-arousal terms, such as ‘mild’ and ‘secure,’ also enhanced liking for the majority of the participants. For the SWEET cluster specifically, the association with ‘secure’ resulted in a significant liking lift of 2.0 scale points. Negative low-arousal terms, such as ‘bored,’ exerted a negative impact on liking, with the SOUR cluster decreasing liking by 1.568 points due to feeling ‘bored.’

Cluster-specific differences were primarily observed in the impacts of high-arousal emotions. Negative high-arousal terms, such as ‘aggressive,’ were associated with a significant liking decrease for the SWEET (−1.807) and IU (−1.365) clusters. However, no such suppression was observed for the SOUR cluster. The impacts of positive high-arousal terms were more variable. Terms such as ‘active,’ ‘adventurous,’ and ‘enthusiastic’ increased liking by at least 1.0 point for the SOUR cluster. In contrast, these terms did not significantly enhance liking for the SWEET and IU clusters. Notably, the SWEET cluster showed a 0.771-point decrease in liking when the term ‘adventurous’ was selected compared to when it was not.

### 3.5 Integrative analysis of the multi-dimensional factors

The preceding sections detailed cluster-specific differences in sensory attributes, conceptual associations, and emotional responses. MFA was used to investigate these drivers of heterogeneous preferences for sweet-sour mixtures. Liking was included as a supplementary variable to evaluate the relationship between these multi-dimensional data sets.

The first two MFA dimensions explained approximately 90% of the total variance for all consumer clusters. High structural consistency was observed between the sensory-conceptual and sensory-emotional blocks (RV > 0.78), indicating a strong coupling between perceptual input and subjective cognitive evaluation. However, the relationships between specific blocks differed substantially across clusters. For the SOUR and SWEET clusters, conceptual associations and emotional responses showed high consistency (RV = 95.2% and 88.1%, respectively). In contrast, the correlation between conceptual and emotional dimensions was weaker for the IU cluster (RV = 62.4%). This suggests that emotional responses in the IU cluster may be driven by specific individual concepts rather than overall conceptual profiles.

**Fig. 8:**
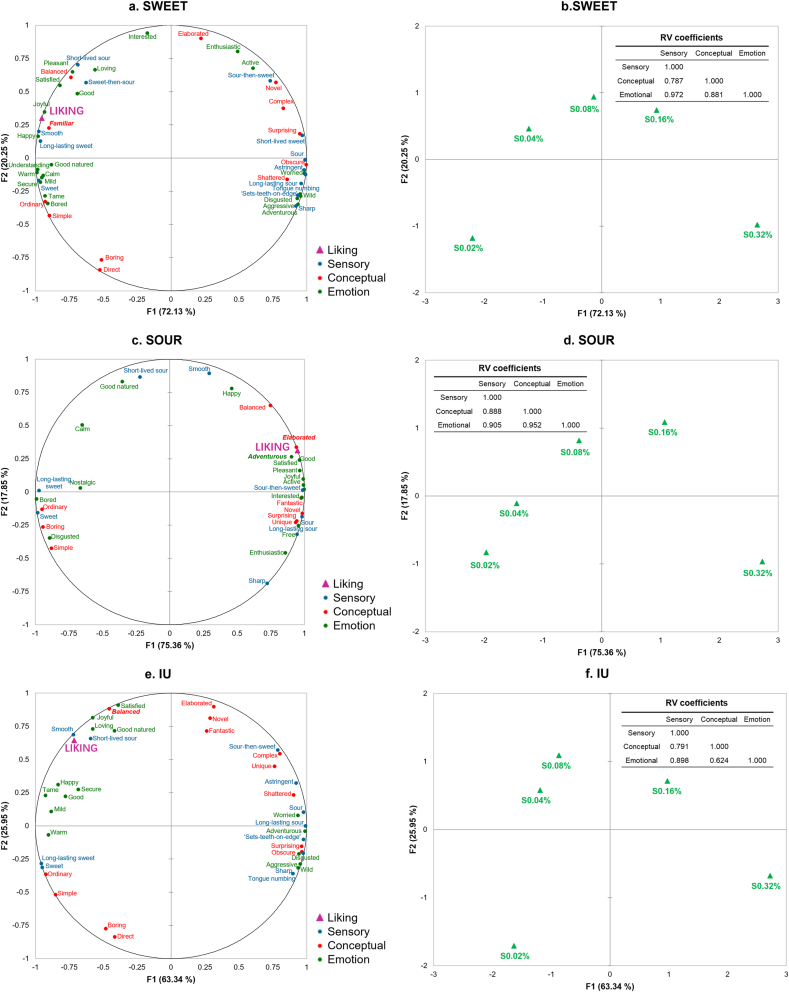
MFA of the five sweet-sour mixtures (S0.02%-S0.32%). Panels illustrate the association among sensory perception, conceptual associations, and emotional responses for the SWEET (a, b), SOUR (c, d), and IU (e, f) clusters based on the first two dimensions. The consensus maps of variables (a, c, e) display sensory attributes (blue), conceptual terms (red), emotional responses (green), and liking (purple triangle). For each cluster, the lower triangle of the matrix provides the RV coefficients calculated using all dimensions of the sensory, conceptual, and emotional data blocks to assess their configurational consistency.

Generally, higher liking scores were associated with positive emotions across all consumer clusters. However, the association patterns among sensory perception, conceptual associations, and emotional responses differed across the identified consumer clusters. Fig. 8 illustrates that the core concepts associated with positive emotions varied among the three clusters. Sensory attributes first formed holistic conceptualizations, which elicited distinct emotional profiles. This process ultimately manifested as the observed heterogeneous preference patterns.

Specifically, for the SWEET cluster, liking was closely associated with the concept of ‘familiar.’ The sensory attributes ‘smooth’ and ‘long-lasting sweet’ were critical in eliciting feelings of ‘familiarity’ and positive emotions. Conversely, attributes such as ‘sour,’ ‘long-lasting sour,’ and ‘sharp’ triggered conceptual associations of ‘obscure,’ ‘shattered,’ and ‘complex.’ These concepts were strongly linked to negative high-arousal emotions.

For the SOUR cluster, liking was strongly linked to sample ‘elaboration.’ Intense sour stimulation provided ‘novel’ and ‘fantastic’ sensory experiences. These perceptions triggered high-arousal emotions, such as ‘adventurous,’ along with other positive emotional responses. Unlike the SWEET cluster, sweetness characteristics for the SOUR cluster overlapped with the concept of ‘simple’ but were correlated with negative emotions, such as ‘disgusted.’

The distribution of sensory and emotional dimensions for the IU cluster partially mirrored that of the SWEET cluster. Sweet-sour balance was the primary driver of liking for IU cluster. Samples exhibiting ‘sweet-then-sour’ transitions were perceived as the most ‘balanced,’ evoking feelings of being ‘satisfied.’ Sourness characteristics were linked to conceptual associations of ‘complex’ and ‘shattered,’ prone to triggering negative emotions.

## 4 Discussion

This research provided a comprehensive understanding of consumer preference heterogeneity in sweet-sour mixtures by evaluating liking alongside sensory, conceptual, and emotional responses within the citric acid-sucrose model system. Four sweet-sour preference patterns were identified: one with a relatively flat intensity-liking function (FLAT) and three with clear variations in hedonic response patterns (SWEET, SOUR, and IU). Our analyses focused on the three clusters with distinct preference patterns (SWEET, SOUR, and IU). When preferences for sourness and sweetness are considered together, consumer heterogeneity remains significant. However, the proportion of consumers or the patterns of preference deviate from the hedonic phenotype observed when only sourness cues are taken into account. Notably, the proportion of sour likers, who show a positive correlation between liking and sourness intensity, was higher than that reported in studies using sour stimuli alone (27.4% in our study vs. 12% in Spinelli et al., 2024). Meanwhile, consumers who did not exhibit a clear sour preference in single-sour studies appeared to differentiate further in the present mixture system, forming two subgroups: one displaying an inverted U-shaped preference pattern and another exhibiting a relatively flat hedonic response.

The variation in cluster distribution suggests that consumers’ preference for sourness is not fixed but may be modulated by the presence of sweetness. On the one hand, suppression of sourness by sucrose may attenuate the sharpness of the acid stimulus (Savant and McDaniel, 2004b). This may enable some consumers who would not have been classified as sour likers in the single-sour stimuli to exhibit a greater preference for sourness in the mixture. On the other hand, this distributional change may also partly reflect differences in the classification criteria used. In previous work, such as that of Spinelli et al. (2024), phenotypes were primarily identified on the basis of a single taste (sourness) intensity-liking relationship. The present study incorporated three correlations, including sourness intensity-liking, sweetness intensity-liking, and sweet/sour ratio-liking. This approach may capture preference structures more effectively in systems where sweetness and sourness coexist.

Furthermore, we sought to verify whether consumers consistently preferred samples with comparable sweet and sour intensities, and whether differences in preference between individuals were attributable to variations in their perceived dominant taste. To this end, we compared the ratings of sourness and sweetness intensity (Section 3.2) and the perceived sensory attributes (Section 3.4.1) across different clusters. The clusters showed consistency in their categorization of the dominance in taste perception (sweet-dominant, comparable sweet-sour intensities, or sour-dominant). This consensus in sensory characterization suggests that preference differentiation does not stem from a divergence in identifying the taste dominance of the stimulus. In other words, consumers do not always prefer samples with comparable sweet and sour intensities. On the contrary, different clusters prefer different sweet/sour ratios.

Comparison of intensity ratings across clusters in the mixture and single-taste systems revealed that the suppressive effect of sourness on sweetness perception was relatively consistent across clusters, whereas the suppressive effect of sweetness on sourness perception was strongly modulated by individual preference phenotypes. Segmentation based on gustatory perception indicates that individual differences in physiological taste sensitivity can influence binary taste interactions (Junge et al., 2020; Prescott et al., 2004). However, in our study, there were no significant differences in the baseline perception of sourness among clusters when testing within the pure citric acid solutions (p > 0.05, Section 3.2). This implies that for different preference clusters, the perceived sourness intensity in the mixture is not solely determined by innate gustatory sensitivity, but may also be related to how sweetness and sourness cues are integrated during the evaluative process. Previous studies have suggested that individual differences in the allocation of attention to taste cues during the processing of complex stimuli may influence the evaluative outcomes (Prescott and Stevenson, 1995; Small and Prescott, 2005). Spinelli et al. (2024) further proposed that sour dislikers may allocate greater attention to sour cues, thereby subjectively amplifying their perceived sourness intensity. Attentional allocation was not directly measured in this study. However, the baseline perception of sourness remained consistent across groups, and participants shared a clear consensus on relative taste dominance. Collectively, these findings imply that the differences in intensity perceptions observed in the sweet-sour mixtures should not be regarded as the primary driver of preference segmentation.

Building on the inference that perceived intensity alone appears insufficient to explain consumer heterogeneity in sweet-sour preferences, we further examined whether these clusters diverge at the subjective cognitive level. Regarding conceptual characteristics, the clusters differed in how they defined ‘sweet-sour balance’ (see Section 3.4.3). In this study, only the IU cluster endorsed the sample with a sweet/sour ratio closest to 1:1 as the most balanced. The SWEET and SOUR clusters judged the most ‘balanced’ samples as those that were sweet-dominant and sour-dominant, respectively. Differences in this conceptual characteristic across clusters suggest that objective perceptual balance and subjective hedonic balance do not necessarily coincide. The former is closer to an intensity-related attribute in the sensory analysis (Liem and de Graaf, 2004; Mao et al., 2025; Martin, 2002; Stolzenbach et al., 2016), whereas the latter reflects whether a consumer perceives a given sweet-sour combination as harmonious and “just right” (Bastian et al., 2010; Koone et al., 2014).

Beyond ‘balance,’ sensory complexity constitutes an additional determinant of preference in mixed-taste systems. Previous research has shown that the relationship between complexity and liking is non-monotonic. Moderate or contextually appropriate complexity can enrich the sensory experience, and optimal complexity levels may vary across consumer segments (Lévy et al., 2006; Spence, 2018). Consistent with this, our findings indicate that the SWEET cluster demonstrated a preference for familiar, simple characteristics, suggesting a bias toward sensory experiences with low cognitive demand. For these consumers, excessive complexity may trigger cognitive overload, thereby reducing liking (Spence, 2020; Wang and Spence, 2019). By contrast, the optimal complexity level for the SOUR cluster may be higher. For these consumers, low-complexity stimuli dominated by a single taste, such as samples S0.02% and S0.04%, may be perceived as monotonous, failing to sustain hedonic engagement (Köster and Mojet, 2007).

Between-cluster divergence was further amplified in the emotional dimension. The SOUR cluster was more likely to associate sour-dominant samples with positive high-arousal emotions such as active, adventurous, and enthusiastic. This suggests that these consumers do not merely tolerate sourness but may regard intense sourness as a novel experience worth exploring. By contrast, positive responses in the SWEET cluster were more strongly linked to familiar sensory experiences. For these consumers, sour-dominant samples were not only less palatable but may also have been associated with greater discomfort or perceived sensory risk. The IU cluster showed less pronounced reliance on any particular emotion type. Their evaluations were more centered on whether the samples achieved sweet-sour balance. These between-cluster differences in emotional response align with established psychological constructs regarding personality traits. Previous research has shown that individuals higher in sensation seeking and reward sensitivity tend to pursue intense and novel sensory experiences, whereas those with greater punishment sensitivity tend to prefer familiar, low-risk, and lower-intensity stimuli (Byrnes and Hayes, 2013, 2016; Spence, 2022; Spinelli et al., 2018). Future studies could examine the relationship between sweet-sour preference phenotypes and personality traits using standardized instruments such as Arnett's Inventory of Sensation Seeking (Arnett, 1994) and the Sensitivity to Punishment and Sensitivity to Reward Questionnaire (Torrubia et al., 2001).

MFA and the corresponding RV coefficients further supported the interpretation of between-cluster differences from a subjective cognitive perspective. Across the three main preference clusters, sensory-conceptual and sensory-emotional data blocks were highly interrelated. This suggests that consumers may not process taste information in isolation but form a coherent evaluative structure integrating sensory perception, conceptual judgment, and emotional response (Giacalone et al., 2022; Ng et al., 2013; Thomson, 2010). However, it is worth noting that consumers differ in the way they integrate information when evaluating sweet-sour mixtures. In the SWEET and SOUR clusters, these data blocks exhibited a high degree of structural congruence. By contrast, the association between the conceptual and emotional dimensions was notably weaker in the IU cluster. In other words, conceptual associations and emotional responses were highly similar in the SWEET and SOUR clusters. The IU cluster, however, appeared to rely more on an analytical evaluation of sweet/sour ratios and balance, rather than on a holistic conceptual-emotional schema. Previous research has demonstrated that cognitive style can significantly influence how consumers perceive and evaluate food products (Beekman et al., 2022; Beekman and Seo, 2024). The between-cluster differences in the coupling of conceptual and emotional response blocks observed here indirectly support this notion. However, the specific cognitive dimensions involved require further investigation through more targeted experimental approaches.

Taken together, the present study addresses the three core questions raised in the Introduction. First, consumers in a sweet-sour mixture system do form distinct preference phenotypes. However, these phenotypes are not simple replications of those identified in single-taste studies. Second, consumers do not consistently prefer samples with comparable sweet and sour intensities, and the between-cluster differences in preference cannot be attributed solely to differences in the perception of taste intensity. Third, different clusters exhibit distinguishable characteristics in their subjective perceptions, which can be summarized as three interpretable patterns in the organization of sensory, conceptual, and emotional preference responses:

(1) The SWEET cluster preferred familiar, simple, and sweet-dominant experiences and exhibited consistently positive conceptual and emotional associations.
(2) The SOUR cluster was more receptive to the novelty and high-arousal experiences associated with strong sourness and assigned more positive emotional meanings to these experiences.
(3) The IU cluster prioritized greater emphasis on overall balance and harmony within the sweet-sour mixture. Their preferences were primarily driven by the sensory equilibrium between sweetness and sourness rather than the dominance of either single taste.

Finally, certain limitations regarding sample representativeness and the stimulus matrix warrant acknowledgment. Convenience sampling was used, and participants consisted primarily of young, highly educated female consumers from Jiangnan University (Wuxi, China). Consequently, the preference phenotypes and their associated psychological organizational patterns primarily reflect the heterogeneity within this specific population. Whether these findings generalize to broader consumer populations remains to be established. Future research should examine the stability of these preference phenotypes in more diverse samples, including greater variation in age, gender, educational level, and cultural background. In addition, the present study used aqueous solution models as stimuli. Although this approach facilitated variable control and enabled systematic characterization of sweet-sour interactions, real food systems typically involve additional sensory cues, including aroma, mouthfeel, and trigeminal stimulation, all of which may influence taste perception (Hewson et al., 2009; Yau and McDaniel, 1992). Extending the preference phenotype framework proposed here to real products, such as fruit juices, carbonated beverages, or fermented drinks, would help establish more application-relevant consumer profiles. It would also allow a more rigorous test of the external validity of these preference patterns in actual consumption contexts.

## 5 Conclusions

The present study confirmed that preference segmentation in binary sweet-sour mixtures is broadly consistent with single-taste hedonic phenotypes, although a higher proportion of sour-preferring consumers was identified. On the basis of intensity-liking correlations, consumers were classified into four clusters. Comprehensive analyses focused on the three clusters with distinct preference phenotypes: the SWEET, SOUR, and IU clusters. Furthermore, applying a multidimensional sensory-conceptual-emotional framework provided deeper insights into sweet-sour preference heterogeneity. The SWEET cluster favored simple and familiar samples, which were associated with positive low-arousal emotional responses and a preference for mild sweetness. By contrast, the SOUR cluster sought intense sour stimulation and showed a greater propensity for novelty-seeking and risk-taking in sensory experience. For the IU cluster, the primary driver of preference was sweet-sour balance, with particular emphasis on the equilibrium between sweetness and sourness intensities. Ultimately, these findings advance our understanding of preference heterogeneity in complex binary taste systems and may facilitate the development of those beverage formulations tailored to the sensory, conceptual, and emotional profiles of specific consumer segments.

## CRediT authorship contribution statement

Jia Chen: Conceptualization, Data curation, Formal analysis, Methodology, Writing - original draft. Feifei Zhao: Conceptualization, Methodology. Fang Zhong: Conceptualization, Methodology, Writing - review & editing. Juntao Kan: Conceptualization, Methodology. Huijuan Shen: Conceptualization, Methodology. Yixun Xia: Conceptualization, Methodology, Writing - review & editing. Charles Spence: Methodology, Writing - review & editing.

## Ethical statement

Ethical approval for the involvement of human participants in this study was granted by the Jiangnan University Medical Ethics Committee (Reference Number: JNU20221201IRB03).

## Declaration of generative AI and AI-assisted technologies in the writing process

During the preparation of this work, the authors used Chat GPT in order to improve the readability and language of the manuscript. After using this tool/service, the authors reviewed and edited the content as needed and take full responsibility for the content of the published article.

## Declaration of competing interest

The authors declare that they have no known competing financial interests or personal relationships that could have appeared to influence the work reported in this paper.

## Data Availability

Data will be made available on request.
