# Supplementary material for: Consumer heterogeneity in sweet-sour preferences: Insights from sensory perception, conceptual associations, and emotional responses
Source: Curr Res Food Sci. 2026 Apr 13;12:101408. doi: 10.1016/j.crfs.2026.101408 (PMC13098337; doi:10.1016/j.crfs.2026.101408)
Supplement: Multimedia component 1 [file mmc1.docx]

**Table S1 Demographic and product usage characteristics of participants (female, n=172)**

| **Characteristic** | **Category** | **Percentage (%)** |
| --- | --- | --- |
| Age (years) | 20–23 | 29.7 |
|  | 24–26 | 63.3 |
|  | 27–34 | 7.0 |
| Consumption Frequency  (products like yogurt or juice) | Once per week | 65.7 |
|  | 3–4 times per week | 18.0 |
|  | Once per day | 16.3 |

Table S2 Frequency percentages (%) of CATA sensory term selection using Cochran’s Q test

| Attributes | S0.02% | S0.04% | S0.08% | S0.16% | S0.32% | p-value (Cochran’s Q) |
| --- | --- | --- | --- | --- | --- | --- |
| **Total Sample** | | | | | | |
| Sweet | 0.952^d^ | 0.780^c^ | 0.583^b^ | 0.321^a^ | 0.208^a^ | < 0.001 |
| Sour | 0.042^a^ | 0.089^a^ | 0.220^b^ | 0.339^c^ | 0.530^d^ | < 0.001 |
| Astringent | 0.030^a^ | 0.036^ab^ | 0.107^bc^ | 0.137^c^ | 0.185^c^ | < 0.001 |
| Bitter | 0.000^a^ | 0.000^a^ | 0.006^a^ | 0.012^a^ | 0.024^a^ | 0.053 |
| Sour-then-sweet | 0.012^a^ | 0.155^b^ | 0.321^c^ | 0.452^c^ | 0.470^c^ | < 0.001 |
| Sweet-then-sour | 0.137^ab^ | 0.250^c^ | 0.220^bc^ | 0.173^abc^ | 0.101^a^ | 0.001 |
| Short-lived sweet | 0.113^ab^ | 0.089^a^ | 0.113^ab^ | 0.167^ab^ | 0.220^b^ | 0.002 |
| Long-lasting sweet | 0.690^c^ | 0.673^c^ | 0.476^b^ | 0.363^b^ | 0.137^a^ | < 0.001 |
| Short-lived sour | 0.185^ab^ | 0.393^c^ | 0.339^c^ | 0.292^bc^ | 0.119^a^ | < 0.001 |
| Long-lasting sour | 0.024^a^ | 0.048^ab^ | 0.119^b^ | 0.268^c^ | 0.536^d^ | < 0.001 |
| Tongue numbing | 0.012^a^ | 0.012^a^ | 0.018^a^ | 0.077^ab^ | 0.155^b^ | < 0.001 |
| ‘Sets-teeth-on-edge' | 0.000^a^ | 0.012^a^ | 0.036^a^ | 0.131^b^ | 0.274^c^ | < 0.001 |
| Sharp | 0.083^a^ | 0.083^a^ | 0.089^a^ | 0.196^a^ | 0.435^b^ | < 0.001 |
| Smooth | 0.256^b^ | 0.333^b^ | 0.315^b^ | 0.268^b^ | 0.065^a^ | < 0.001 |
| Bland | 0.077^b^ | 0.018^ab^ | 0.018^ab^ | 0.006^a^ | 0.006^a^ | < 0.001 |
| **SWEET** | | | | | | |
| Sweet | 0.984^d^ | 0.787^c^ | 0.508^b^ | 0.213^a^ | 0.098^a^ | < 0.001 |
| Sour | 0.049^a^ | 0.115^ab^ | 0.262^bc^ | 0.475^cd^ | 0.623^d^ | < 0.001 |
| Astringent | 0.049^a^ | 0.049^a^ | 0.164^ab^ | 0.213^ab^ | 0.328^b^ | < 0.001 |
| Sour-then-sweet | 0.016^a^ | 0.148^ab^ | 0.361^bc^ | 0.525^c^ | 0.328^bc^ | < 0.001 |
| Sweet-then-sour | 0.131^ab^ | 0.344^b^ | 0.180^b^ | 0.148^ab^ | 0.016^a^ | < 0.001 |
| Short-lived sweet | 0.082^a^ | 0.131^a^ | 0.180^a^ | 0.262^a^ | 0.279^a^ | 0.019 |
| Long-lasting sweet | 0.639^cd^ | 0.705^d^ | 0.443^bc^ | 0.279^b^ | 0.066^a^ | < 0.001 |
| Short-lived sour | 0.230^ab^ | 0.311^b^ | 0.344^b^ | 0.230^ab^ | 0.049^a^ | 0.001 |
| Long-lasting sour | 0.016^a^ | 0.082^ab^ | 0.213^bc^ | 0.377^c^ | 0.639^d^ | < 0.001 |
| Tongue numbing | 0.000^a^ | 0.016^a^ | 0.033^a^ | 0.148^ab^ | 0.230^b^ | < 0.001 |
| ‘Sets-teeth-on-edge' | 0.000^a^ | 0.000^a^ | 0.016^a^ | 0.230^b^ | 0.393^b^ | < 0.001 |
| Sharp | 0.066^a^ | 0.115^a^ | 0.131^a^ | 0.246^a^ | 0.623^b^ | < 0.001 |
| Smooth | 0.410^b^ | 0.377^b^ | 0.328^b^ | 0.180^b^ | 0.000^a^ | < 0.001 |
| **SOUR** | | | | | | |
| Sweet | 0.913^c^ | 0.783^c^ | 0.652^bc^ | 0.370^ab^ | 0.261^a^ | < 0.001 |
| Sour | 0.000^a^ | 0.022^a^ | 0.087^a^ | 0.196^ab^ | 0.413^b^ | < 0.001 |
| Astringent | 0.000^a^ | 0.000^a^ | 0.022^a^ | 0.022^a^ | 0.022^a^ | 0.736 |
| Sour-then-sweet | 0.000^a^ | 0.152^ab^ | 0.261^b^ | 0.391^bc^ | 0.630^c^ | < 0.001 |
| Sweet-then-sour | 0.109^a^ | 0.087^a^ | 0.196^a^ | 0.152^a^ | 0.109^a^ | 0.525 |
| Short-lived sweet | 0.109^a^ | 0.043^a^ | 0.087^a^ | 0.065^a^ | 0.174^a^ | 0.208 |
| Long-lasting sweet | 0.739^b^ | 0.696^b^ | 0.478^ab^ | 0.457^ab^ | 0.196^a^ | < 0.001 |
| Short-lived sour | 0.174^a^ | 0.326^a^ | 0.283^a^ | 0.370^a^ | 0.130^a^ | 0.018 |
| Long-lasting sour | 0.022^a^ | 0.022^a^ | 0.043^a^ | 0.196^ab^ | 0.478^b^ | < 0.001 |
| Tongue numbing | 0.022^a^ | 0.022^a^ | 0.022^a^ | 0.043^a^ | 0.043^a^ | 0.921 |
| ‘Sets-teeth-on-edge' | 0.000^a^ | 0.022^a^ | 0.043^a^ | 0.043^a^ | 0.087^a^ | 0.092 |
| Sharp | 0.109^a^ | 0.087^a^ | 0.065^a^ | 0.087^a^ | 0.261^a^ | 0.021 |
| Smooth | 0.130^a^ | 0.217^ab^ | 0.261^ab^ | 0.413^b^ | 0.174^ab^ | 0.005 |
| **IU** | | | | | | |
| Sweet | 0.951^d^ | 0.732^cd^ | 0.610^bc^ | 0.366^ab^ | 0.220^a^ | < 0.001 |
| Sour | 0.049^a^ | 0.122^ab^ | 0.244^ab^ | 0.341^bc^ | 0.585^c^ | < 0.001 |
| Astringent | 0.024^a^ | 0.073^a^ | 0.098^a^ | 0.195^a^ | 0.195^a^ | 0.012 |
| Sour-then-sweet | 0.024^a^ | 0.171^ab^ | 0.317^b^ | 0.439^b^ | 0.390^b^ | < 0.001 |
| Sweet-then-sour | 0.195^a^ | 0.220^a^ | 0.293^a^ | 0.220^a^ | 0.195^a^ | 0.772 |
| Short-lived sweet | 0.122^a^ | 0.049^a^ | 0.073^a^ | 0.171^a^ | 0.195^a^ | 0.115 |
| Long-lasting sweet | 0.756^c^ | 0.537^bc^ | 0.512^bc^ | 0.293^ab^ | 0.146^a^ | < 0.001 |
| Short-lived sour | 0.195^a^ | 0.512^b^ | 0.341^ab^ | 0.293^ab^ | 0.098^a^ | < 0.001 |
| Long-lasting sour | 0.024^a^ | 0.024^ab^ | 0.122^ab^ | 0.268^bc^ | 0.439^c^ | < 0.001 |
| Tongue numbing | 0.024^ab^ | 0.000^a^ | 0.000^a^ | 0.049^ab^ | 0.244^b^ | < 0.001 |
| ‘Sets-teeth-on-edge' | 0.000^a^ | 0.024^a^ | 0.049^a^ | 0.122^ab^ | 0.317^b^ | < 0.001 |
| Sharp | 0.073^a^ | 0.073^a^ | 0.049^a^ | 0.244^ab^ | 0.463^b^ | < 0.001 |
| Smooth | 0.171^ab^ | 0.390^b^ | 0.415^b^ | 0.220^ab^ | 0.000^a^ | < 0.001 |

^a,b,c,d^ indicate significant differences between samples for each attribute. Post hoc pairwise comparisons analysis was conducted using McNemar’s test.

Table S3 Frequency percentages (%) of CATA conceptual term selection using Cochran’s Q test

| Attributes | S0.02% | S0.04% | S0.08% | S0.16% | S0.32% | p-value (Cochran’s Q) |
| --- | --- | --- | --- | --- | --- | --- |
| **SWEET** | | | | | | |
| Novel | 0.049^a^ | 0.180^ab^ | 0.180^ab^ | 0.246^b^ | 0.213^ab^ | 0.017 |
| Ordinary | 0.361^c^ | 0.262^bc^ | 0.098^ab^ | 0.082^ab^ | 0.033^a^ | < 0.001 |
| Surprising | 0.000 | 0.148^ab^ | 0.213^b^ | 0.213^b^ | 0.344^b^ | < 0.001 |
| Boring | 0.197^a^ | 0.049^a^ | 0.066^a^ | 0.049^a^ | 0.082^a^ | 0.018 |
| Fantastic | 0.115^a^ | 0.279^a^ | 0.262^a^ | 0.279^a^ | 0.197^a^ | 0.069 |
| Simple | 0.508^c^ | 0.311^bc^ | 0.131^ab^ | 0.098^ab^ | 0.066^a^ | < 0.001 |
| Complex | 0.016^a^ | 0.131^ab^ | 0.328^b^ | 0.230^b^ | 0.328^b^ | < 0.001 |
| Direct | 0.475^b^ | 0.279^ab^ | 0.197^a^ | 0.213^a^ | 0.295^ab^ | 0.003 |
| Obscure | 0.033^a^ | 0.115^ab^ | 0.262^bc^ | 0.344^cd^ | 0.574^d^ | < 0.001 |
| Balanced | 0.213^b^ | 0.328^b^ | 0.328^b^ | 0.164^ab^ | 0.016^a^ | < 0.001 |
| Shattered | 0.049^a^ | 0.164^ab^ | 0.148^ab^ | 0.131^ab^ | 0.328^b^ | < 0.001 |
| Short-lived | 0.213^a^ | 0.180^a^ | 0.082^a^ | 0.164^a^ | 0.098^a^ | 0.132 |
| Elaborated | 0.115^a^ | 0.213^a^ | 0.279^a^ | 0.344^a^ | 0.148^a^ | 0.005 |
| Unique | 0.098^a^ | 0.131^a^ | 0.213^a^ | 0.279^a^ | 0.197^a^ | 0.064 |
| Familiar | 0.311^b^ | 0.262^b^ | 0.213^ab^ | 0.246^b^ | 0.049^a^ | 0.002 |
| **SOUR** | | | | | | |
| Novel | 0.043^a^ | 0.065^a^ | 0.109^a^ | 0.239^ab^ | 0.413^b^ | < 0.001 |
| Ordinary | 0.457^b^ | 0.370^b^ | 0.174^ab^ | 0.174^ab^ | 0.000^a^ | < 0.001 |
| Surprising | 0.043^a^ | 0.152^ab^ | 0.109^a^ | 0.239^ab^ | 0.435^b^ | < 0.001 |
| Boring | 0.391^c^ | 0.283^bc^ | 0.130^abc^ | 0.087^ab^ | 0.000^a^ | < 0.001 |
| Fantastic | 0.043^a^ | 0.109^ab^ | 0.109^ab^ | 0.304^bc^ | 0.413^c^ | < 0.001 |
| Simple | 0.283^a^ | 0.217^a^ | 0.174^a^ | 0.043^a^ | 0.087^a^ | 0.016 |
| Complex | 0.065^a^ | 0.087^a^ | 0.130^a^ | 0.217^a^ | 0.196^a^ | 0.121 |
| Direct | 0.413^a^ | 0.217^a^ | 0.239^a^ | 0.196^a^ | 0.239^a^ | 0.103 |
| Obscure | 0.130^a^ | 0.152^a^ | 0.239^a^ | 0.130^a^ | 0.174^a^ | 0.527 |
| Balanced | 0.022^a^ | 0.152^ab^ | 0.261^ab^ | 0.348^b^ | 0.261^b^ | 0.001 |
| Shattered | 0.109^a^ | 0.152^a^ | 0.152^a^ | 0.130^a^ | 0.000^a^ | 0.069 |
| Short-lived | 0.174^a^ | 0.109^a^ | 0.152^a^ | 0.152^a^ | 0.065^a^ | 0.364 |
| Elaborated | 0.022^a^ | 0.109^ab^ | 0.283^bc^ | 0.413^c^ | 0.435^c^ | < 0.001 |
| Unique | 0.065^a^ | 0.065^a^ | 0.043^a^ | 0.239^ab^ | 0.370^b^ | < 0.001 |
| Familiar | 0.152^a^ | 0.196^a^ | 0.196^a^ | 0.152^a^ | 0.152^a^ | 0.937 |
| **IU** | | | | | | |
| Novel | 0.098^a^ | 0.146^a^ | 0.268^a^ | 0.268^a^ | 0.171^a^ | 0.033 |
| Ordinary | 0.415^b^ | 0.244^ab^ | 0.244^ab^ | 0.073^a^ | 0.024^a^ | < 0.001 |
| Surprising | 0.049^a^ | 0.098^a^ | 0.073^a^ | 0.195^ab^ | 0.463^b^ | < 0.001 |
| Boring | 0.244^a^ | 0.024^a^ | 0.073^a^ | 0.049^a^ | 0.049^a^ | 0.001 |
| Fantastic | 0.098^a^ | 0.366^a^ | 0.244^a^ | 0.268^a^ | 0.268^a^ | 0.022 |
| Simple | 0.512^c^ | 0.268^bc^ | 0.171^abc^ | 0.073^ab^ | 0.000^a^ | < 0.001 |
| Complex | 0.049^a^ | 0.195^ab^ | 0.293^ab^ | 0.293^ab^ | 0.366^b^ | 0.004 |
| Direct | 0.585^b^ | 0.317^ab^ | 0.171^a^ | 0.293^ab^ | 0.268^ab^ | 0.002 |
| Obscure | 0.122^a^ | 0.098^a^ | 0.146^ab^ | 0.220^ab^ | 0.415^b^ | 0.001 |
| Balanced | 0.098^ab^ | 0.268^ab^ | 0.341^b^ | 0.244^ab^ | 0.049^a^ | 0.005 |
| Shattered | 0.000^a^ | 0.049^a^ | 0.049^a^ | 0.220^a^ | 0.195^a^ | 0.001 |
| Short-lived | 0.073^a^ | 0.171^a^ | 0.098^a^ | 0.098^a^ | 0.073^a^ | 0.480 |
| Elaborated | 0.024^a^ | 0.220^ab^ | 0.390^b^ | 0.415^b^ | 0.220^ab^ | < 0.001 |
| Unique | 0.073^a^ | 0.268^a^ | 0.171^a^ | 0.293^a^ | 0.317^a^ | 0.034 |
| Familiar | 0.317^a^ | 0.146^a^ | 0.244^a^ | 0.171^a^ | 0.098^a^ | 0.099 |

^a,b,c,d^ indicate significant differences between samples for each term. Post hoc pairwise comparisons analysis was conducted using McNemar’s test.

Table S4 Frequency percentages (%) of CATA emotional term selection using Cochran’s Q test

| Attributes | S0.02% | S0.04% | S0.08% | S0.16% | S0.32% | p-value (Cochran’s Q) |
| --- | --- | --- | --- | --- | --- | --- |
| **SWEET** | | | | | | |
| Warm | 0.295^c^ | 0.246^bc^ | 0.148^abc^ | 0.082^ab^ | 0.016^a^ | < 0.001 |
| Bored | 0.279^b^ | 0.148^ab^ | 0.098^ab^ | 0.098^ab^ | 0.033^a^ | < 0.001 |
| Disgusted | 0.049^a^ | 0.049^a^ | 0.115^ab^ | 0.131^ab^ | 0.279^b^ | < 0.001 |
| Nostalgic | 0.131^a^ | 0.115^a^ | 0.033^a^ | 0.066^a^ | 0.033^a^ | 0.057 |
| understanding, | 0.213^b^ | 0.131^ab^ | 0.082^ab^ | 0.131^ab^ | 0.000^a^ | 0.003 |
| Wild | 0.016^a^ | 0.033^a^ | 0.082^ab^ | 0.279^b^ | 0.689^c^ | < 0.001 |
| Good natured | 0.230^b^ | 0.115^ab^ | 0.131^ab^ | 0.066^ab^ | 0.000^a^ | 0.001 |
| Joyful | 0.262^b^ | 0.279^b^ | 0.246^b^ | 0.148^ab^ | 0.000^a^ | < 0.001 |
| Interested | 0.098^ab^ | 0.180^ab^ | 0.230^ab^ | 0.246^b^ | 0.049^a^ | 0.003 |
| Happy | 0.279^b^ | 0.262^b^ | 0.180^b^ | 0.148^ab^ | 0.000^a^ | < 0.001 |
| Free | 0.098^a^ | 0.180^a^ | 0.197^a^ | 0.164^a^ | 0.098^a^ | 0.303 |
| Guilty | 0.016^a^ | 0.000^a^ | 0.033^a^ | 0.016^a^ | 0.033^a^ | 0.675 |
| Mild | 0.344^b^ | 0.295^b^ | 0.131^ab^ | 0.049^a^ | 0.000^a^ | < 0.001 |
| Pleasant | 0.148^ab^ | 0.164^b^ | 0.197^b^ | 0.148^ab^ | 0.000^a^ | 0.006 |
| Satisfied | 0.148^ab^ | 0.164^b^ | 0.180^b^ | 0.115^ab^ | 0.000^a^ | 0.011 |
| Tame | 0.311^c^ | 0.246^bc^ | 0.066^ab^ | 0.049^a^ | 0.000^a^ | < 0.001 |
| Loving | 0.049^ab^ | 0.164^b^ | 0.131^ab^ | 0.049^ab^ | 0.000^a^ | 0.001 |
| Secure | 0.262^b^ | 0.230^b^ | 0.082^ab^ | 0.082^ab^ | 0.000^a^ | < 0.001 |
| Active | 0.049^a^ | 0.230^ab^ | 0.197^ab^ | 0.311^b^ | 0.213^ab^ | 0.005 |
| Enthusiastic | 0.066^a^ | 0.197^ab^ | 0.246^ab^ | 0.344^b^ | 0.180^ab^ | 0.002 |
| Worried | 0.033^a^ | 0.033^a^ | 0.164^ab^ | 0.230^b^ | 0.377^b^ | < 0.001 |
| Aggressive | 0.016^a^ | 0.049^ab^ | 0.115^ab^ | 0.213^b^ | 0.508^c^ | < 0.001 |
| Calm | 0.344^c^ | 0.295^c^ | 0.180^bc^ | 0.066^ab^ | 0.000^a^ | < 0.001 |
| Adventurous | 0.016^a^ | 0.098^ab^ | 0.066^ab^ | 0.246^b^ | 0.541^c^ | < 0.001 |
| Good | 0.197^ab^ | 0.180^ab^ | 0.279^b^ | 0.131^ab^ | 0.049^a^ | 0.003 |
| **SOUR** | | | | | | |
| Warm | 0.130^a^ | 0.174^a^ | 0.130^a^ | 0.152^a^ | 0.043^a^ | 0.292 |
| Bored | 0.326^b^ | 0.326^b^ | 0.196^ab^ | 0.109^ab^ | 0.000^a^ | < 0.001 |
| Disgusted | 0.217^a^ | 0.217^a^ | 0.065^a^ | 0.043^a^ | 0.022^a^ | 0.001 |
| Nostalgic | 0.109^a^ | 0.217^a^ | 0.109^a^ | 0.087^a^ | 0.065^a^ | 0.034 |
| understanding, | 0.087^a^ | 0.087^a^ | 0.174^a^ | 0.217^a^ | 0.152^a^ | 0.186 |
| Wild | 0.130^a^ | 0.109^a^ | 0.043^a^ | 0.109^a^ | 0.087^a^ | 0.608 |
| Good natured | 0.087^a^ | 0.087^a^ | 0.196^a^ | 0.130^a^ | 0.022^a^ | 0.032 |
| Joyful | 0.043^a^ | 0.109^ab^ | 0.152^ab^ | 0.239^ab^ | 0.326^b^ | 0.002 |
| Interested | 0.022^a^ | 0.065^ab^ | 0.087^ab^ | 0.283^bc^ | 0.391^c^ | < 0.001 |
| Happy | 0.065^a^ | 0.043^a^ | 0.239^a^ | 0.239^a^ | 0.130^a^ | 0.003 |
| Free | 0.043^a^ | 0.065^a^ | 0.087^a^ | 0.109^a^ | 0.217^a^ | 0.046 |
| Guilty | 0.043^a^ | 0.043^a^ | 0.022^a^ | 0.000^a^ | 0.000^a^ | 0.349 |
| Mild | 0.261^a^ | 0.152^a^ | 0.239^a^ | 0.174^a^ | 0.109^a^ | 0.213 |
| Pleasant | 0.022^a^ | 0.065^ab^ | 0.152^ab^ | 0.217^ab^ | 0.304^b^ | 0.001 |
| Satisfied | 0.022^a^ | 0.022^a^ | 0.217^ab^ | 0.217^ab^ | 0.283^b^ | < 0.001 |
| Tame | 0.130^a^ | 0.152^a^ | 0.196^a^ | 0.174^a^ | 0.043^a^ | 0.184 |
| Loving | 0.022^a^ | 0.043^a^ | 0.087^a^ | 0.087^a^ | 0.174^a^ | 0.076 |
| Secure | 0.065^a^ | 0.109^a^ | 0.109^a^ | 0.152^a^ | 0.043^a^ | 0.346 |
| Active | 0.065^a^ | 0.087^a^ | 0.174^ab^ | 0.304^ab^ | 0.413^b^ | < 0.001 |
| Enthusiastic | 0.109^a^ | 0.087^a^ | 0.065^a^ | 0.152^a^ | 0.261^a^ | 0.026 |
| Worried | 0.109^a^ | 0.152^a^ | 0.065^a^ | 0.065^a^ | 0.022^a^ | 0.203 |
| Aggressive | 0.065^a^ | 0.043^a^ | 0.065^a^ | 0.087^a^ | 0.196^a^ | 0.053 |
| Calm | 0.261^ab^ | 0.196^ab^ | 0.413^b^ | 0.174^ab^ | 0.022^a^ | < 0.001 |
| Adventurous | 0.000^a^ | 0.043^ab^ | 0.130^ab^ | 0.174^ab^ | 0.217^b^ | 0.001 |
| Good | 0.043^ab^ | 0.022^a^ | 0.152^abc^ | 0.261^bc^ | 0.304^c^ | < 0.001 |
|  |  |  | **IU** |  |  |  |
| Warm | 0.244^a^ | 0.293^a^ | 0.146^a^ | 0.098^a^ | 0.024^a^ | 0.005 |
| Bored | 0.220^a^ | 0.122^a^ | 0.122^a^ | 0.024^a^ | 0.122^a^ | 0.064 |
| Disgusted | 0.073^a^ | 0.098^a^ | 0.049^a^ | 0.146^a^ | 0.244^a^ | 0.029 |
| Nostalgic | 0.098^a^ | 0.049^a^ | 0.024^a^ | 0.073^a^ | 0.049^a^ | 0.548 |
| understanding, | 0.220^a^ | 0.122^a^ | 0.122^a^ | 0.195^a^ | 0.073^a^ | 0.255 |
| Wild | 0.098^a^ | 0.073^a^ | 0.024^a^ | 0.220^ab^ | 0.537^b^ | < 0.001 |
| Good natured | 0.073^a^ | 0.098^a^ | 0.171^a^ | 0.171^a^ | 0.000^a^ | 0.041 |
| Joyful | 0.122^ab^ | 0.293^b^ | 0.317^b^ | 0.220^ab^ | 0.049^a^ | 0.004 |
| Interested | 0.073^a^ | 0.195^a^ | 0.293^a^ | 0.195^a^ | 0.122^a^ | 0.075 |
| Happy | 0.195^ab^ | 0.341^b^ | 0.220^ab^ | 0.122^ab^ | 0.049^a^ | 0.002 |
| Free | 0.122^a^ | 0.146^a^ | 0.171^a^ | 0.171^a^ | 0.098^a^ | 0.756 |
| Guilty | 0.000^a^ | 0.000^a^ | 0.000^a^ | 0.000^a^ | 0.024^a^ | 0.406 |
| Mild | 0.220^ab^ | 0.146^ab^ | 0.268^b^ | 0.073^ab^ | 0.000^a^ | 0.002 |
| Pleasant | 0.098^a^ | 0.244^a^ | 0.220^a^ | 0.195^a^ | 0.122^a^ | 0.182 |
| Satisfied | 0.073^a^ | 0.195^a^ | 0.244^a^ | 0.195^a^ | 0.049^a^ | 0.009 |
| Tame | 0.195^ab^ | 0.171^ab^ | 0.244^b^ | 0.098^ab^ | 0.000^a^ | 0.007 |
| Loving | 0.024^a^ | 0.171^a^ | 0.171^a^ | 0.049^a^ | 0.000^a^ | 0.006 |
| Secure | 0.146^ab^ | 0.122^ab^ | 0.293^b^ | 0.024^a^ | 0.024^a^ | < 0.001 |
| Active | 0.220^a^ | 0.195^a^ | 0.220^a^ | 0.195^a^ | 0.293^a^ | 0.788 |
| Enthusiastic | 0.268^a^ | 0.195^a^ | 0.146^a^ | 0.171^a^ | 0.049^a^ | 0.054 |
| Worried | 0.000^a^ | 0.000^a^ | 0.024^a^ | 0.171^a^ | 0.171^a^ | < 0.001 |
| Aggressive | 0.049^a^ | 0.000^a^ | 0.024^a^ | 0.146^ab^ | 0.366^b^ | < 0.001 |
| Calm | 0.220^a^ | 0.098^a^ | 0.195^a^ | 0.098^a^ | 0.049^a^ | 0.072 |
| Adventurous | 0.073^a^ | 0.073^a^ | 0.171^a^ | 0.293^ab^ | 0.512^b^ | < 0.001 |
| Good | 0.171^ab^ | 0.293^b^ | 0.146^ab^ | 0.122^ab^ | 0.049^a^ | 0.005 |

^a,b,c,d^ indicate significant differences between samples for each term. Post hoc pairwise comparisons analysis was conducted using McNemar’s test.
